# Monitoring the Activation of a AuCu Aerogel CO_2_-Reduction Electrocatalyst via *Operando* XAS

**DOI:** 10.1021/acs.langmuir.5c00662

**Published:** 2025-04-25

**Authors:** Maximilian Winzely, Adam H. Clark, Deema Balalta, Piyush Chauhan, Paul M. Leidinger, Meriem Fikry, Tym de Wild, Maximilian Georgi, Alexander Eychmüller, Sara Bals, Thomas J. Schmidt, Juan Herranz

**Affiliations:** † PSI, Center for Energy and Environmental Science, CH-5232 Villigen, Switzerland; ‡ PSI, Center for Photon Science, CH-5232 Villigen, Switzerland; § University of Antwerp Electron Microscopy for Materials Science, BE-2020 Antwerpen, Belgium; ∥ Technische Universität Dresden, Physical Chemistry, DE-01062 Dresden, Germany; ⊥ ETH Zürich, Institute for Molecular Physical Science, CH-8093 Zürich, Switzerland

## Abstract

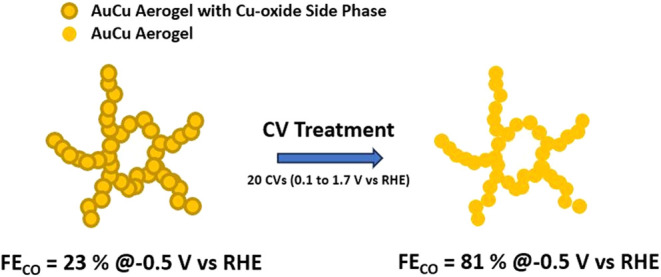

The electrochemical reduction of CO_2_ is a promising approach to mitigate global warming by converting CO_2_ into valuable industrial chemicals such as CO. Among the various CO_2_-electroreduction catalysts investigated, AuCu alloys have proven to be particularly promising as they exhibit even higher activity and selectivity toward CO production compared to pure Au, which can be considered as one of the state-of-the-art catalysts for this reaction. In a recent study, we showed that unsupported AuCu aerogels feature an appealing CO_2_-to-CO activity and selectivity, even if in their as-synthesized form they were not phase-pure but instead contained Cu oxide. Thus, in this work, we aim at understanding how the transformation of this bimetallic and compositionally heterogeneous aerogel induced by a cyclic voltammetry (CV) treatment leads to this enhanced CO_2_-electroreduction performance. This was done by applying three different experimental protocols, implying (i) the absence of this CV treatment, (ii) the completion of the CV treatment without exchanging the electrolyte prior to the CO_2_-reduction test, or (iii) the CV treatment and exchanging the electrolyte before performing the CO_2_-reduction potential hold. These three protocols were complemented with *operando* grazing incidence X-ray absorption spectroscopy (GIXAS) measurements that revealed the structural and compositional changes undergone by the AuCu aerogel during CV treatment. The latter is then shown to lead to the removal of Cu oxide side phases and the enrichment of the aerogel’s surface with Au atoms and a AuCu alloy phase, which in turn results in a significant increase in the faradaic efficiency toward CO, from 23 to 81% when this CV treatment is overlooked vs performed, respectively.

## Introduction

Climate change is accelerating at an unprecedented rate, underlining the urgent need for the rapid development and implementation of technologies for the efficient reuse of carbon.^1^ Among the many approaches currently being explored, the electrochemical reduction of CO_2_ stands out as a particularly promising technique.^2,3^ However, to fully exploit the potential of this approach, catalysts with high selectivity and activity at low overpotentials are needed. In this context, the electrochemical production of carbon monoxide (CO) or formate has been predicted to be economically viable, rendering both of these products particularly attractive.^4−7^

So far, mostly precious metals such as gold (Au) and silver (Ag) have proven to be active and selective catalysts for CO production.^8−10^ Nevertheless, further progress is needed to increase their mass activity, which is crucial for improving the overall efficiency and cost-effectiveness of catalysts in industrial applications. Specifically, improving the mass activity implies that less catalyst is needed to achieve the same level of performance, reducing material costs or energy consumption. For Au, one approach to do so is by lowering the surface adsorption strength toward CO. This goal can possibly be achieved by changing the gold’s electronic structure through alloying with other metals.^11−15^ Thus, in an effort to improve the CO_2_-to-CO activity and selectivity, AuCu alloys have emerged as particularly promising candidates.^16−21^ In this context, one study has shown that structurally ordered AuCu nanoparticles with a Au-rich surface featured a faradaic efficiency (FE) toward CO of ≈80% at −0.77 V vs the reversible hydrogen electrode (RHE), while the disordered nanoparticle counterpart had a Cu-rich surface favoring hydrogen evolution.^16^ Another study introduced a AuCu core–shell catalyst with the Au atoms mostly exposed on the surface and exhibiting an FE for CO of ≈94% at −0.8 V vs RHE, along with a superior mass activity for CO of ≈440 mA/mg_Au_ at the same potential.^18^ However, in several of the above works,^18−20^ these bimetallic nanoparticles were dispersed on carbon-based supports that are known to catalyze the undesired evolution of H_2_^22,23^ at the large overpotentials concomitant to the high current densities (≥200 mA cm^–2^) that have to be attained to render CO_2_-electroreduction industrially relevant.^2,7,24^ To address this issue, a recent study in our group^21^ explored the use of an unsupported AuCu aerogel as a CO_2_ reduction electrocatalyst whereby also other aerogel catalysts have previously demonstrated high selectivity and activity for CO production in CO_2_ electroreduction, making them promising alternatives to conventional supported catalysts.^21,23,25−27^ In this study, we have observed a significant influence of a cyclic voltammetry (CV) treatment on its CO_2_ reduction reaction (CO_2_RR) selectivity and activity. More precisely, when the catalyst was tested with or without this pretreatment, it achieved an FE for CO of 87 vs 34%, respectively, and when the CV-treated sample was benchmarked against a monometallic Au aerogel, it featured a 2-fold increase in the Au-mass-normalized activity.^28^ While identical location transmission electron microscopy (IL-TEM) and electrochemical measurements showed that the CV treatment caused the partial removal of Cu-based oxide side phases and qualitative changes in the aerogel’s surface composition, further characterization by more sensitive techniques was missing to fully understand the atomic and electronic structural changes (e.g., (de)alloying extent, surface vs bulk composition) caused by the CV treatment and tie them to the concomitant activity and selectivity enhancements caused by it. Thus, the main objective of this study is to investigate the electronic and structural modifications occurring in the AuCu aerogel during CV treatment and to better understand how these changes contribute to enhanced catalytic performance. By gaining deeper insights into the structure–activity relationship, we aim to identify the key factors responsible for the improved selectivity and activity toward CO. To achieve this, we employed a combination of operando X-ray absorption spectroscopy (XAS) and electrochemical characterization to bridge this knowledge gap and provide a comprehensive picture of the catalyst’s transformations.

Since the changes undergone by the catalyst during these CVs are likely dependent on the transport of Cu-derived ionic species produced during the treatment, and thus on the convective properties of the electrochemical cell used for these tests, we have upgraded the cell^29^ utilized for CO_2_RR experiments in the previous study^21^ to enable *operando* XAS investigations without modifying these mass transport features.^30^ Chiefly, this modification leverages a grazing incidence (GI) geometry^30^ that facilitates the study of these dynamic changes using time-resolved, quick-scanning XAS (QuickXAS) while keeping the very low catalyst loading of only 100 μg_catalyst_/cm^2^ used in the CO_2_RR tests featured in our previous work.^21^ This assures that our results are unaffected by artifacts stemming from the use of excessively thick catalyst layers concomitant to the highly loaded electrodes that are generally needed when performing XAS measurements in non-GI acquisition geometries, like the accumulation of gas bubbles or the appearance of potential gradients along the catalyst layer’s thickness.^31,32^ As will be shown below, the *operando* GIXA-spectra acquired with this new spectroelectrochemical cell unveiled that the CV treatment results in the removal of Cu oxide side phases and the enrichment of the aerogel’s surface with Au, which in turn lead to its enhanced CO_2_RR-performance.

## Experimental Section

### Aerogel Synthesis

The synthesis procedure closely followed the method described in ref (33). In summary, the exact amounts of HAuCl_4_·3H_2_O (99.99%, abcr GmbH) and CuCl_2_ (99.995%, Fischer Scientific) were dissolved in 400 mL of ethanol (99 + 1% petroleum ether, Berkel AHK) to achieve concentrations of 0.1 mM of both chemicals for the synthesis of the AuCu aerogel. The solution was degassed with N_2_ (5.0, ALPHAGAZ) and stirred at 450 rpm for 30 min to prevent oxidation. A 50 mM stock solution of NaBH_4_ was added rapidly with bubbling and stirring until a concentration of 6 equiv was reached. The reaction mixture was allowed to stand for 1–2 days until gelation occurred, resulting in the settling of the aggregated gel fragments of the nanoparticles. These settled gels were washed thoroughly with ethanol seven times over 3 days before being transferred to an autoclave, where the solvent was replaced by CO_2_ and a supercritical drying process at 37 °C and 90 bar was followed to obtain pulverized aerogels.

### Electrode and Electrolyte Preparation

For the preparation of the electrodes, the AuCu aerogel catalyst was deposited by drop-casting onto 35 μm thick graphene sheets (Nanografi) using the following ink formulation. About 4 mg of aerogel was carefully weighed into a vial, and then one part of isopropanol (Sigma-Aldrich, HPLC grade, 99.9%) and three parts of ultrapure water (18.2 MΩ·cm, Elga PureLab) were added successively. In addition, Na^+^-exchanged Nafion dispersion^34^ was added to the ink in a weight ratio of 10% with regard to the aerogel’s mass. The volume of the ink was adjusted so that an aerogel loading of 100 μg/cm^2^ was achieved when the droplet volume was set to 50 μL. The drop-casting of the electrodes was performed after the ink was sonicated for 1 min. To ensure precise positioning and shape of the catalyst layer on the graphene substrate, the same mask as introduced in ref (29) was used.

A 250 mL volumetric flask was used to prepare all of the electrolyte solutions. The phosphate buffer, which was used to calibrate the Ag/AgCl reference electrode (Innovative Instruments, Inc.) against the reversible hydrogen electrode (*vide infra*), was prepared by dissolving 1.872 g of dipotassium hydrogen phosphate (K_2_HPO_4_, Merck LiChropur, anhydrous, 99.999%) and 1.939 g of potassium dihydrogen phosphate (KH_2_PO_4_, Merck, LiChropur, anhydrous, 99.999%) in ultrapure water to achieve a concentration of 0.1 M and a pH of about 6.82. As for the 0.5 M KHCO_3_ solution used for the CO_2_-reduction measurements, 12.515 g of the bicarbonate salt (99.95% trace metal base, Sigma-Aldrich) was dissolved again in 250 mL of ultrapure water.

### Spectroelectrochemical Cell

**Figure 1:**
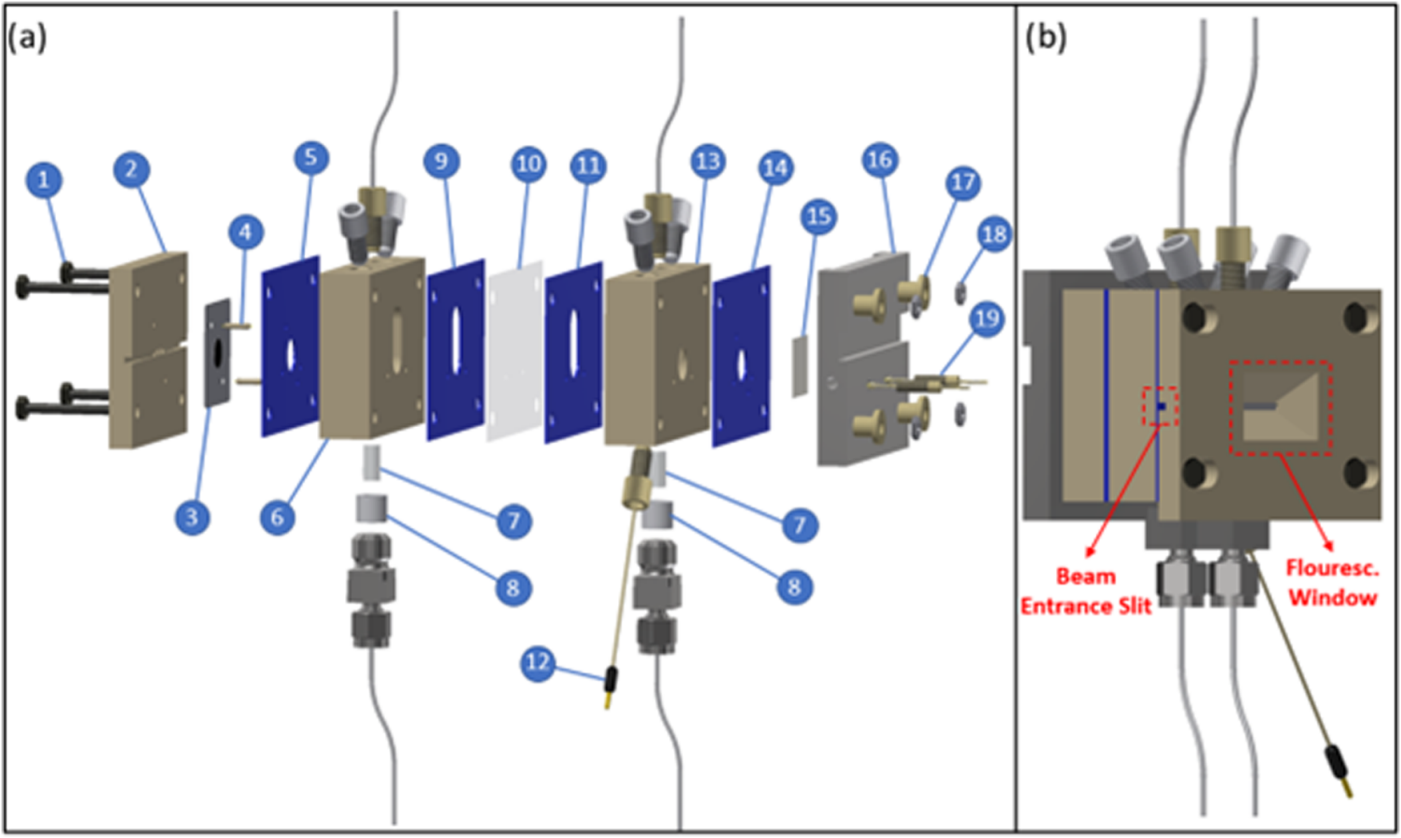
(a) Technical drawing of the spectroeletrochemical GIXAS cell including four M5 screws (1), the working electrode part (2), graphene foil with catalyst (3), alignment pins (4), ice cube gaskets (5, 9, 11, 14), the working electrode compartment (6), porous glass frits (7), PTFE frit holders (8), Nafion XL membrane (10), leak-free Ag/AgCl reference electrode (12), the counter electrode compartment (13), platinum counter electrode (15), the counter electrode current collector (16), four PEEK inserts (17), four M5 nuts (18), and two gold pins inserted into PEEK screws (19). (b) Side view of the GIXAS cell for better illustration of the beam entrance slit and fluorescence window.

The spectroelectrochemical cell, as shown in Figure 1, was used for all of the subsequent experiments discussed herein. This design is based on the *online* gas chromatography electrochemical cell described in ref (29) and was modified to make the *operando* GIXAS analysis of the working electrode possible. Therefore, a similar design as in ref (30) was chosen, allowing the incident X-ray beam to hit the electrode at a grazing angle <1°. Two slits were milled into the working electrode part (2) so that the X-ray beam first hits the electrode from behind before probing the catalyst, which is facing the electrolyte compartment on the other side. Furthermore, a window was machined into this part to facilitate the detection of the fluorescence signal (Figure 1b). Two gold pins (18) are used to contact the working electrode, which consists of a 35 μm thick graphene sheet (Nanografi) and the desired catalyst (3). These pins, which are attached to the current collector of the counter electrode (16) by PEEK screws, run through the entire cell. In contrast to the PTFE gaskets formerly used to seal the cell described in ref (29), 0.8 mm thick ice cube gaskets (FC-PO100, Freudenberg–5, 9, 11, 14) were applied herein, since the latter provide better sealing properties when tightening the four M5 screws (1) by hand. To detect and reliably quantify the CO_2_RR-products, the working electrode compartment is separated from the counter electrode compartment by a membrane (Chemours, Nafion XL–13). All of the other features of this cell remain the same as already described in ref (29).

### Cyclic Voltammetry (CV) Treatment

In order to investigate the effect of the CV treatment on the selectivity and activity of the aerogel for the electrochemical reduction of CO_2_, an experimental protocol consisting of 20 cycles from 0.1 to 1.7 V vs RHE (with the first cycle starting upward and from the open circuit voltage (OCV)) at 50 mV/s followed by one more cycle at a scan rate of 20 mV/s in the same potential range. This approach contrasts with the five cycles used by Chauhan et al.;^21^ the additional cycles were necessary in this work to achieve a stable current profile for the last CV cycles of this CV treatment. During this electrochemical procedure, a flow rate of 4.5 and 3 sccm CO_2_ (5.3, PanGas) was bubbled through the working- and counter electrode compartments, respectively, to maintain CO_2_ saturation of the electrolyte.

In total, three different experimental procedures were performed. In the first one, referred to as “no CV treatment”, the catalyst was not subjected to any potential cycling prior to the CO_2_ reduction potential hold. In the “CV treatment” and “CV treatment + EE” experimental protocols, the aerogel underwent the previously described potential cycling treatment. The main difference lies in the latter protocol (whereby “EE” stands for “electrolyte exchange”), in which the electrolyte was removed from both compartments of the spectroelectrochemical cell and rinsed thoroughly eight times with ultrapure water before a fresh electrolyte was added. This additional step was intended to prevent the deposition on the catalyst’s surface of any ions (e.g., Cu^2+^) that could have been stripped from the catalyst during the CV cycling during the subsequent CO_2_RR potential hold. Notably, whereas in the “CV treatment + EE” experiment in ref (21) the CV treatment was performed in a separate glass cell prior to the potential hold in the *online* GC cell, all CV treatments described here were performed within the custom-built spectroelectrochemical cell.

### CO_2_-Electroreduction Potential Holds

The potential holds were performed in the above-mentioned spectroelectrochemical cell using a Ag/AgCl reference electrode and a platinum foil (Alfa Aesar, 99.99%) as the counter electrode with a CO_2_-saturated 0.5 M KHCO_3_ as the electrolyte (*vide supra*). Prior to this, the reference electrode was calibrated by hydrogen evolution/oxidation experiments performed on a polycrystalline platinum rotating disk electrode at a rotational speed of 1600 rpm. A 0.1 M phosphate buffer solution with a pH of 6.82 was used for this calibration (see above). The actual potential shift for the CO_2_-saturated 0.5 M KHCO_3_ was calculated based on a pH value of 7.28.

A constant flow of 4.5 and 3 sccm CO_2_ was continuously bubbled through the working and counter electrode compartments for the entire duration of each potential hold. To quantify the gaseous products, the outlet of the working compartment was connected directly to a gas chromatograph (SRI Instruments, 8610 C). Each potential was maintained for a period of 60 min, with gas chromatograph injections every 7.5 min. After each experiment, the electrolyte was extracted from the working electrode chamber and an aliquot of it was analyzed for the formate content by ion chromatography (Metrohm, 882 Compact IC plus). The analysis of other liquid-phase products, such as alcohols, was not performed, as a previous study with the same AuCu aerogel found no detectable amounts of these species.^21^

The potential during the experiments was controlled with a VMP-3 potentiostat in the laboratory and an SP-300 potentiostat for synchrotron experiments, both from BioLogic. First, an impedance spectrum was recorded at OCV with a perturbation of 10 mV to determine the high-frequency resistance from the Nyquist plot, which repeatedly resulted in values between 55 and 60 Ω·cm^2^. All potentials were then corrected for 85% of the determined resistance. Linear sweep voltammetry (LSV) was used to scan at a rate of 20 mV/s from the OCV to the holding potential at −500 mV vs RHE. After holding at −500 mV vs RHE for 60 min, an oxidative linear sweep up to 1.7 V vs RHE was performed, followed by CVs ranging from 0.1 to 1.7 V vs RHE, using again a scan rate of 20 mV/s.

### *Operando* XAS

*Operando* XAS experiments were performed at the Super-XAS beamline (X10DA) of the Swiss Light Source (SLS).^35^ The XA-spectra were recorded at the Cu K-edge (8978.9 keV) and the Au L_3_-edge (11918.7 keV) simultaneously in fluorescence mode during the CV treatment and potential holds. The polychromatic beam, collimated by a Pt-coated mirror at 2.84 mrad, was generated by a 2.9 T superbend magnetic source. A Si(111) channel fast scanning monochromator with liquid N_2_ cooling was used to produce the monochromatic beam. A Pt-coated toroidal mirror focused the beam to a spot size of 0.15 × 0.15 mm^2^. The beam flux interacting with the sample was 5 × 1111 photons/s. Three identical ionization chambers (15 cm long, filled with 2 bar N_2_) were used to measure the intensity of the incident beam as a function of energy (in front of the sample) and the XAS signal of a piece of Au foil, used as an energy reference, which was placed in front of a third ionization chamber. Fluorescence detection was performed in fast XAS mode with QuickXAS by using a PIPS diode detector from Mirion Technology at a monochromator oscillation of 1 Hz.^36^ Vertical, horizontal, and angular scans were performed sequentially to align the cell with the sample in the GI geometry. This iterative process was continued until the highest Cu Kα fluorescence signal was recorded at the SDD detector for all samples.

Data processing and analysis were performed using ProQEXAFS^37^ and Demeter software.^38^ The extended X-ray absorption fine structure (EXAFS) spectra were Fourier-transformed in the k range from 3 to 12 or 3 to 8.5 k^–1^ for the Au L_3_-edge and from 3 to 10 or 3 to 8.3 k^–1^ for the Cu K-edge depending on the data quality. The crystal parameters for Au (ICSD-52700), Cu (ICSD-136042), CuO (ICSD-16025), and AuCu alloy (ICSD-42574) required for the fitting were extracted from crystal structures obtained from the ICSD database for inorganic crystal structures. The amplitude reduction factors for all k-ranges were determined by fitting the spectrum of the Au and Cu reference foils, which were used for energy calibration with a fixed coordination number of 12 (Figures S1 and S2 and Tables S1 and S2).

For the CV treatment, the data analysis was performed as follows: after extracting the raw data with ProQEXAFS, six spectra were averaged before normalization, resulting in a temporal resolution of 3 s, since two spectra per second were recorded at a monochromator oscillation of 1 Hz. This can also be translated into a potential resolution of 150 and 60 mV per data point for scan rates of 50 and 20 mV/s, respectively. For both the averaged Cu K-edge and Au L_3_-edge spectra, SIMPLISMA-derived components were used to initialize a further analysis with multivariate curve resolution (MCR)^39^ and the resulting component spectra were used for EXAFS fitting to identify the chemical nature of the components.^40−42^

### Scanning Transmission Electron Microscopy (STEM)

High-angle annular dark-field scanning transmission electron microscopy (HAADF STEM) and energy-dispersive X-ray spectroscopy (EDS) were acquired using a ThermoFisher Tecnai Osiris Microscope operated at 200 kV, equipped with a Super X EDS detector. EDS measurements were performed at a beam current of 50 pA. The sample preparation involved dispersing the AuCu aerogel in a mixture of isopropanol and Milli-Q water (25:75 by volume) by sonicating for 1 min. The suspension was then drop-casted onto a lacy carbon TEM grid for imaging.

## Results and Discussion

### Potential Cycling

As mentioned already in the experimental part, 20 CVs had to be recorded instead of the 5 cycles used by Chauhan et al.^21^ (reproduced in Figure S3) in order to achieve a stable current profile for the last CVs. To determine whether the need for these additional cycles could be tied to a difference in the initial state of the catalyst powder used here as compared to the one featured in ref (21), possibly stemming from the aging of the material, we performed HAADF STEM coupled with EDS on the AuCu aerogel. The acquired HAADF STEM images and corresponding EDS elemental maps are displayed in Figure S4 of the Supporting Information, and revealed regions of varying contrast, with bright areas surrounded by lower-contrast regions, that the EDS analysis confirmed to correspond to Au-rich domains vs Cu oxide phases, respectively (in the latter case, owing to the overlapping signals for Cu and O). This suggests a heterogeneous composition within the aerogel structure that is consistent with what was reported in our previous study^21^ for the same AuCu aerogel when it was processed into an ink (hypothetically due to Cu-segregation and oxidation upon ultrasonic treatment). However, in that same work, the as-synthesized powder did not feature such a high amount of Cu oxide side phases as what is observed here. Thus, it can be hypothesized that this increase in the concentration of Cu oxide side phases in the as-synthesized aerogel is primarily the result of its prolonged atmospheric exposure.

**Figure 2:**
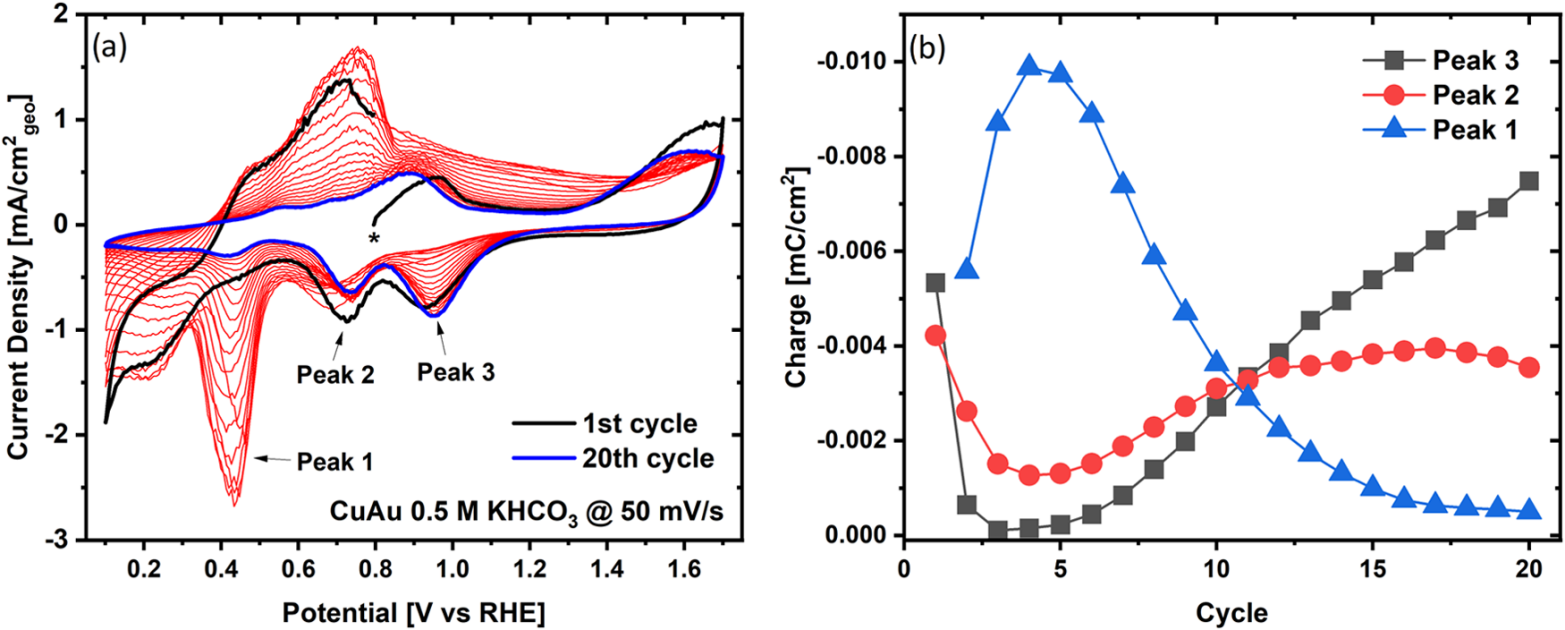
(a) CVs recorded during the *operando* XAS measurements of the CV treatment of a 100 μg_catalyst_/cm^2^ AuCu aerogel working electrode at a scan rate of 50 mV/s in a CO_2_-saturated 0.5 M KHCO_3_ between 0.1 and 1.7 V vs RHE. The first cycle (start marked by a star) of the CV treatment is illustrated as a black line, while the last cycle is depicted as a blue line. (b) Evolution of the charge of the CVs’ reductive peaks over the cycle number, whereby peak 1 describes the charge for electroplating of Cu ions, peak 2 depicts the charge for the reduction of the AuCu alloy oxide, and peak 3 describes the charge associated with Au oxide reduction.

Moving on to the voltammetric treatment, the features of the CVs displayed in Figure 2a can be tied to the surface changes that occur during the CV treatment of the AuCu aerogel. To improve clarity and facilitate interpretation, we have integrated the charge associated with the three reductive peaks labeled in the figure (see Figure S5 for an example of how this was done), which resulted in the charge vs cycle number plot featured in Figure 2b. The charge associated with the most prominent peak (Peak 1), at ≈0.42 V vs RHE, corresponds to the electrochemical deposition of Cu ions from the electrolyte.^43,44^ The second peak, observed at ≈0.75 V vs RHE, represents the reductive charge of the redox couple of the AuCu alloy phase, with its oxidative counterpart appearing at 0.9 V vs RHE during the positive-going scan.^45^ This peak should not be attributed to the reduction of a Au hydroxide phase, as it remains visible even when the CV limits are set between 0.1 and 1.2 V vs RHE, where no Au-oxidation should occur (see Figure S6). Finally, the third peak at ≈0.95 V vs RHE is related to the reduction of Au oxide. In what follows, the evolution of these charges for all three peaks is discussed in greater detail and complemented by the results derived from the *operando* XAS measurement.

**Figure 3:**
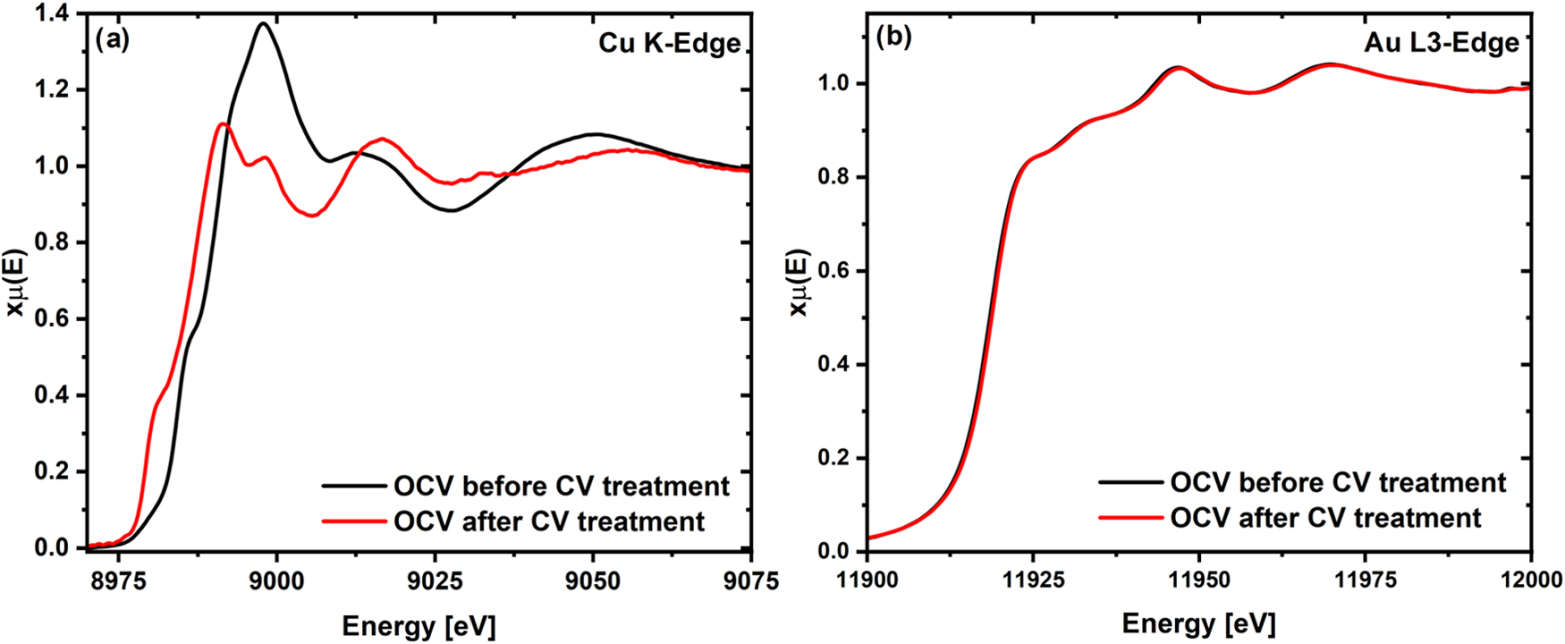
Comparison of the *operando* XAS spectrum at the (a) Cu K-edge and at the (b) Au L3-edge at the OCV before and after the CV treatment of a AuCu aerogel working electrode with a loading of 100 μg_catalyst_/cm^2^ in CO_2_-saturated 0.5 M KHCO_3_. Each spectrum represents an acquisition time of 150 s.

The *operando*, Cu K-edge XA-spectra collected at the OCV before and after performing the CV treatment reveal a significant change in the state of the Cu atoms within the catalyst (see Figure 3a). For example, the shift in the edge position to lower energies indicates that Cu within the catalyst was reduced during the CV treatment. In contrast, the complementary *operando* spectra at the Au L_3_-edge, acquired under the same conditions, show minimal spectral differences before and after the CV treatment (see Figure 3b).

**Figure 4:**
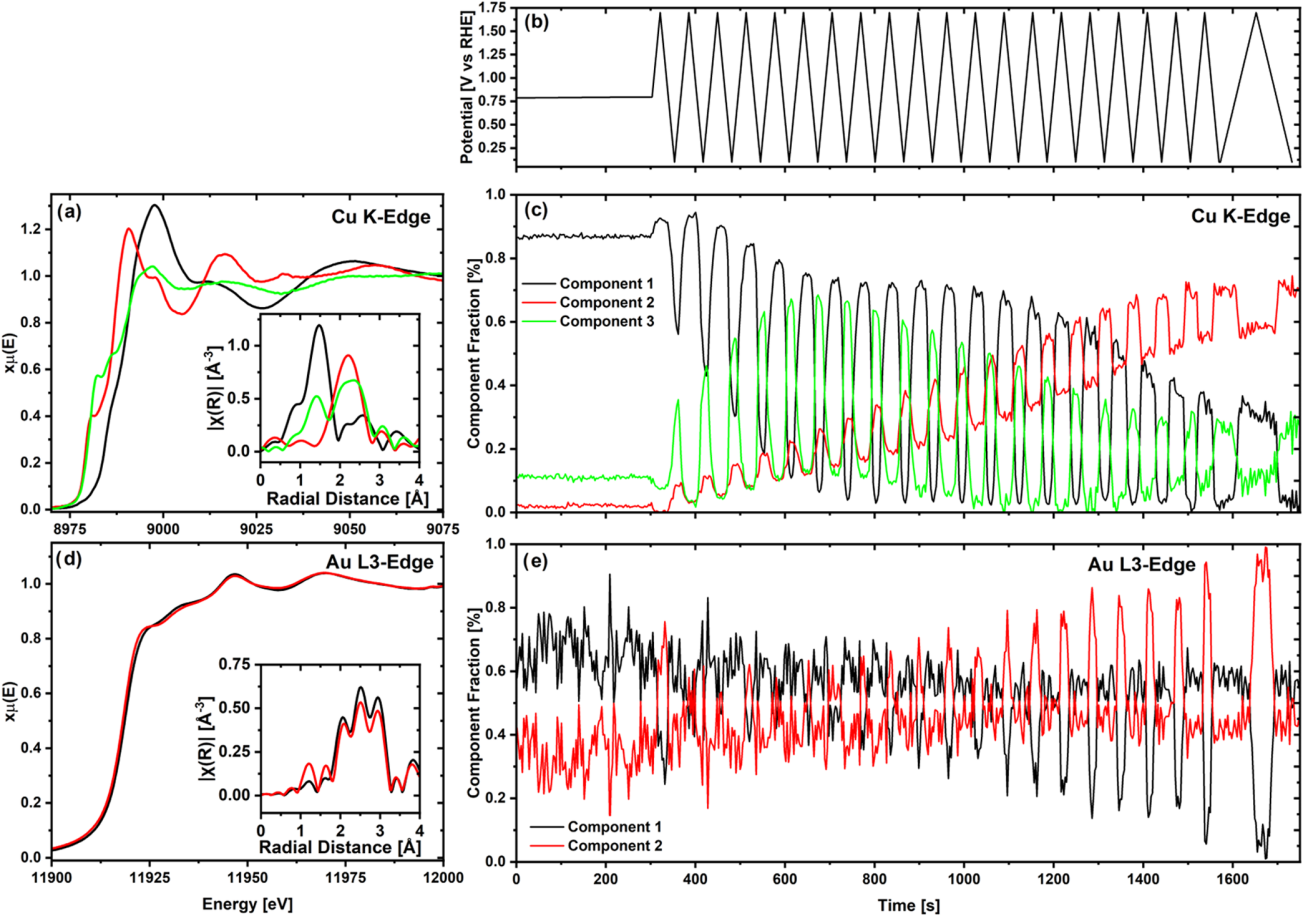
Results of the *operando* GIXAS measurement of a AuCu electrode with a loading of 100 μg_catalyst_/cm^2^ in a CO_2_-saturated 0.5 M KHCO_3_. Applied potential during the CV treatment (b) and multivariant curve resolution analysis of the spectra collected at the Cu K-edge (c) and the Au L_3_-edge (e) using the XA-spectra of the corresponding components in panels (a, d), respectively. The Fourier-transformed EXAFS spectra are shown as insets for all components in panel (a) for the Cu K-edge and in panel (d) for the Au L_3_-edge.

Given the (initially) heterogeneous composition of the AuCu aerogel discussed above, these XA-spectra should be constituted by different contributions of these components (e.g., metallic vs oxidized Cu for the Cu K-edge spectra). Thus, to understand how this transformation of the AuCu catalyst proceeds throughout the CV treatment while differentiating these phases, the entire *operando* XA-spectra acquired during the CV treatment were submitted to an MCR analysis^39^ (described in the Experimental Section) that yields the minimum number of spectral components needed to represent the whole data set. The results of this analysis are shown in Figure 4, in which three distinct components can be identified to describe the entire set of spectra acquired at the Cu K-edge. The first component, identified through EXAFS fitting (see Figure S7), corresponds primarily to a nonstoichiometric Cu(II) oxide phase (Figure S8a). This phase is undercoordinated with oxygen, with Cu exhibiting a low coordination number (CN) for metallic bonding to Au, measured at 0.9 ± 0.2 (Table S3). This observation suggests the presence of Cu oxide islands on Au, as supported by the HAADF STEM images (Figure S4). In addition, component 2 is assigned to a AuCu alloy phase in which Cu has a CN of 6.9 ± 1.4 to Cu and 2.7 ± 0.5 to Au based on the EXAFS fitting of the MCR spectrum (see Figure S7 and Table S3). Finally, component 3 was identified as a Cu(I) oxide phase (see Figure S8b), in which Cu has no observable coordination with Au (cf. Table S3).

Two very similar components were identified through the MCR analysis of the spectra acquired at the Au L_3_ edge, where only a slight shift in the XANES to negative energies can be seen for component 2 compared to that for component 1 (see Figure 4d). Specifically, following the EXAFS fitting of the spectra derived from this analysis (displayed in Figure S9), component 1 was found to have a low coordination number (CN) of 0.7 ± 0.1 with respect to Cu and 8.8 ± 1.0 with respect to Au, while for component 2, the CN was 0.5 ± 0.1 with respect to Cu and 7.3 ± 1.0 with respect to Au (see Table S4). We note in passing that during the fitting, it was not possible for either of these components to reach a good agreement between fit and experimental data when the first-shell path corresponding to the scattering between Au and oxygen atoms was included. However, when scaled by a factor of ≈11.25, the difference between the two components’ spectra is almost identical to the difference between the standard spectra of metallic Au and Au(III)oxide (see Figure S10), thus implying that component 2 is slightly more oxidized than component 1.

Having identified the spectral components derived from the MCR, we now discuss the evolution of their concentrations in the course of the CV treatment, featured in Figure 4c,e, together with the evolution of the charges for the three peaks in the CVs (Figure 2). The *operando* XAS data at the OCV (≈0.8 V vs RHE) indicate that in its initial state the catalyst primarily consists of separate Cu oxide and Au phases, with only a small fraction of Au and Cu atoms present as a AuCu alloy. This is complemented by the surface-sensitive CVs in Figure 2a, in which the first voltammogram features large charges assignable to the oxidation and reduction of Au and AuCu, indicating that both of these phases are already present on the aerogel’s surface in its initial state. This is also endorsed by the Au L_3_ edge data, in which the more oxidized AuCu alloy phase (“component 2”—*vide supra*) becomes dominant during the first cycle to high potentials, which triggers the electrochemical oxidation of the Au surface atoms. Interestingly, a close-to-negligible current peak related to Cu deposition (i.e., peak 3 in Figure 2a) was featured in the CV during the first cycle, suggesting that no significant amount of Cu was dissolved into the electrolyte during the first oxidative scan from OCV to 1.7 V vs RHE.

**Figure 5:**
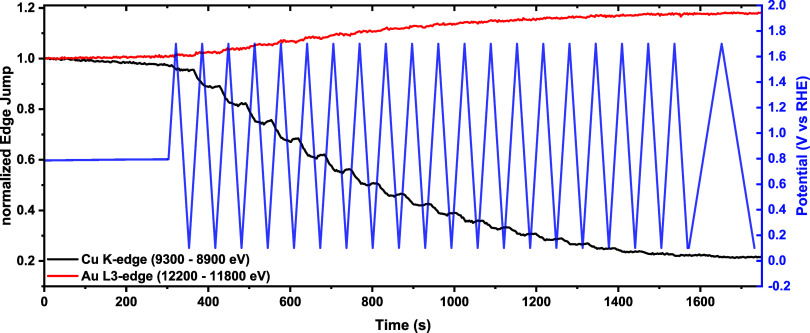
*Operando* normalized Cu K-edge (black line) and Au L_3_-edge jump (red line) during the CV treatment of a AuCu working electrode with a loading of 100 μg_catalyst_/cm^2^ in a CO_2_-saturated 0.5 M KHCO_3_ where one data point corresponds to an average of 6 spectra. The potential variation over time is depicted as a blue line.

In the subsequent potential cycles, a large oxidative current peaking at ≈0.7 V vs RHE was observed during the positive-going scans (see Figure 2a). This can be attributed to the oxidation and dissolution of copper in the electrolyte,^43,44^ which is in turn tied to the increasing charge of peak 3 in Figure 2, associated with the deposition of Cu ions, which were stripped off the catalyst’s surface during the oxidative part of each potential scan, which was already qualitatively observed by Chauhan et al.^21^ through identical location TEM. Furthermore, this behavior is confirmed by the edge jump heights of both the Cu K and Au L_3_ edge spectra, which are proportional to the amount of each element sampled by the X-ray beam^46^ and appear normalized with regard to their values in the initial OCV hold in Figure 5. More precisely, for each recorded CV, the excursions to increasingly positive potentials are associated with a decrease in the Cu K-edge jump indicative of copper dissolution, while when the lower potentials are reached, a slight increase in the Cu concentration is observed due to the replating of a part of the dissolved Cu on the aerogel’s surface. Beyond these potential-driven changes, the overall concentration of the aerogel’s Cu content decreases by ≈80% of its initial value during the complete CV treatment, whereas the edge jump height of the Au L_3_ edge increases by ≈20%. This last increase could be caused by the exposition of Au atoms caused by the dissolution of Cu atoms during the CV treatment that would otherwise absorb the fluorescence photons emitted by these “shielded” Au atoms.

Interestingly, this explanation is also supported by the decrease in the charge associated with the Au- and AuCu-reduction processes (peaks 1 and 2 in Figure 2, respectively) observed during the first four cycles of the CV treatment. More precisely, we hypothesize that this behavior can result from the plating on the aerogel’s Au and AuCu surface atoms of the Cu that gets oxidized in these first cycles, as indicated by the concomitant increase in the normalized edge jump of the Cu K-edge observed in the lower potential (i.e., Cu^x+^-reductive) sections of the CVs featured in Figure 5. However, as the number of cycles keeps on progressing, the charge associated with the reduction of the Au oxide and AuCu phases (i.e., peaks 1 and 2 in Figure 2, respectively) increases again, pointing at an enhanced presence of Au atoms and AuCu domains on the aerogel’s surface. This interpretation is supported by the decrease of the Cu deposition charge (peak 3 in Figure 2) and the Cu-dissolution rate (Figure 5), which indicate that as the CV treatment advances, an increasingly larger fraction of the Cu ions accumulated near the aerogel’s surface diffuse into the bulk of the electrolyte instead of redepositing on the catalyst. Notably, this interpretation is confirmed by XAS data at the Au L_3_-edge, in which the concentration of the more-oxidizable gold-based component (number 2 in Figure 4d,e) increases with the cycle number, indicating that more Au atoms prone to electrochemical oxidation at positive potentials accumulate at the sample’s surface.

Complementarily, the Cu K-edge data unambiguously indicate that the AuCu alloy phase associated with component 2 in Figure 4a–c becomes the dominant phase during the CV treatment. Since this was not accompanied by significant changes at the Au L_3_-edge, it is likely that this AuCu alloy phase was not newly formed during the potential cycling but that instead became the most prominent phase at the Cu K-edge due to the dissolution of Cu oxide side phases. This is again endorsed by the evolution of the Cu K-edge jump in Figure 5, from which one infers that ≈80% of the initial copper inventory was oxidized and dissolved into the electrolyte during the CV treatment, supporting the conclusion that this removal of Cu allowed for the AuCu alloy phase to emerge as the dominant copper-based species.

In summary, the combined electrochemical and *operando* XAS data show that the CV treatment leads to the removal of Cu oxide side phases at high oxidative potentials. This in turn results in an enrichment of the aerogel’s surface with Au atoms and a AuCu alloy phase, additionally indicating that the Cu atoms in the aerogel that are closely coordinated to Au atoms are capable of withstanding this CV treatment.

### Potential Hold

After shedding light on the effects of the CV treatment on the surface and bulk composition of the AuCu aerogel catalyst, we investigated the impact of those CV-induced changes on the activity and selectivity of the resulting material for the electrochemical reduction of CO_2_ in 0.5 M KHCO_3_. The potential holds were performed using the exact same spectroelectrochemical cell and experimental conditions applied during the *operando* XAS measurements, but in addition, the cathode outlet was connected to a gas chromatograph to detect and quantify the gaseous reaction products online. Three different potential holds were carried out for 60 min at −500 mV vs RHE by scanning down from OCV to the holding potential at a scan rate of 20 mV/s. Thereby, each potential hold involves a different initial condition of the catalyst, as already explained in the Experimental Section.

**Figure 6:**
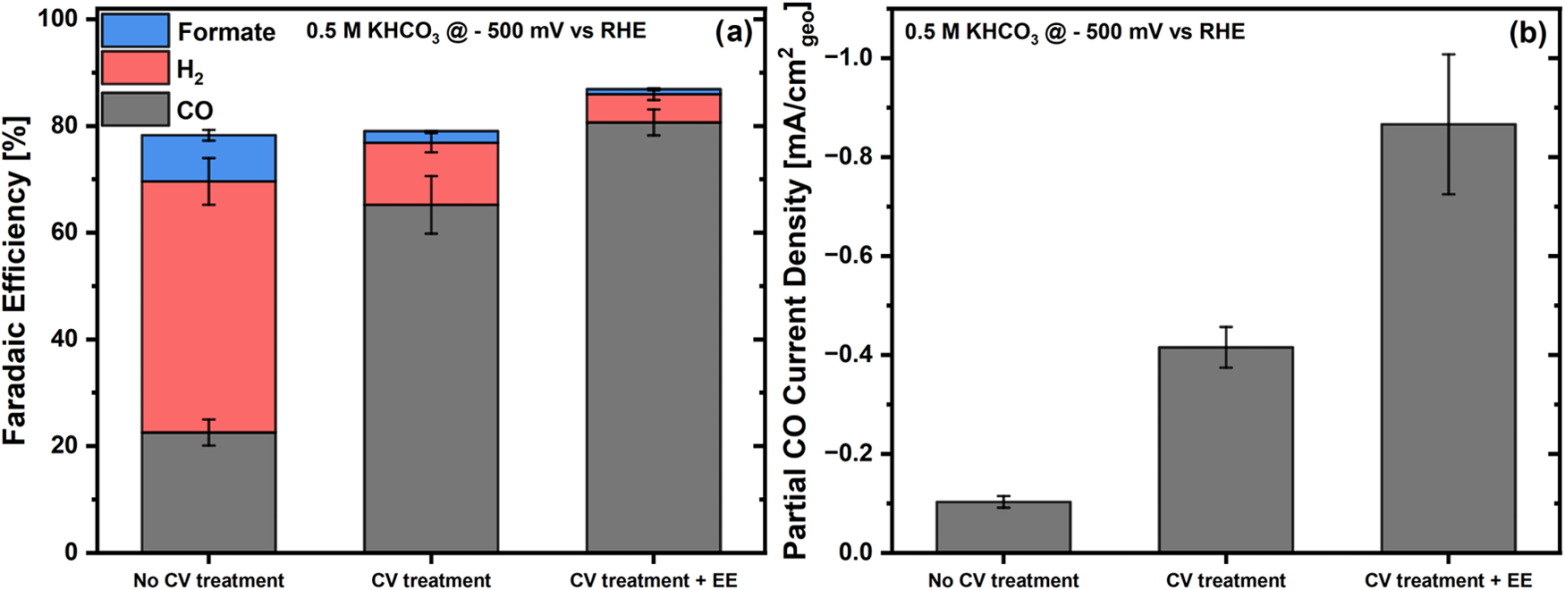
(a) FEs for CO, H_2_, and formate production and (b) partial CDs for CO during the electrochemical reduction of CO_2_ in a CO_2_-saturated 0.5 M KHCO_3_ for a 100 μg_catalyst_/cm^2^ AuCu aerogel working electrode undergoing no CV treatment, CV treatment, or CV treatment + EE prior to the potential hold at −0.5 V vs RHE.

The results of the CO_2_-electroreduction selectivity and activity measurements are featured in Figure 6 and reveal that regardless of whether the electrolyte was exchanged or not, the CV treatment systematically led to a higher FE for CO. Specifically, the CV treatment with EE achieved an FE of 81%, while the CV treatment without EE achieved 65%, whereas performing no CV treatment resulted in a much lower CO FE of only 23% that probably stems from the Cu-rich surface of the aerogel in its initial state (*vide supra*). Notably, this is consistent with the similar CO_2_RR performances observed for this non-CV-treated material and the monometallic Cu-aerogel tested by Chauhan et al. (21), which also featured a high selectivity toward hydrogen at this potential.

As for the increased CO selectivity observed for the CV-treated electrodes, we hypothesize that this can be attributed to the oxidative stripping of the Cu oxide side phases and enrichment of the surface with Au atoms during the CV treatment discussed in the previous section. In the specific case of the “CV treatment + EE” experiment, despite the higher degree of oxidation of the initial aerogel catalyst compared to our previous study (see HAADF STEM images in Figure S4 and the discussion above), an FE toward CO of 81% was achieved, whereas this selectivity was 91% in ref (21) (see Figure S11). Considering that standard deviations of ±10% are customary in such FE values,^29^ such values can be regarded as being in close agreement, thus implying that as long as the CV treatment is adapted to attain similar voltammetric features for the treated samples (see Figures 2a vs S3), a similar product selectivity can be reached during the potential hold.

Interestingly, whereas Chauhan et al.^21^ reported similar CO FEs for the CV-treated samples with or without electrolyte exchange, herein the FE for CO was significantly lower for the “CV treatment” measurement as compared to that for the “CV treatment + EE” counterpart (65 vs 81%, respectively). This may be caused by the larger concentration of Cu oxide side phases in the aerogel used herein vs the one in ref (21) (discussed above), which should result in a larger amount of Cu dissolved in the electrolyte and thus likely to redeposit on the aerogel’s surface and influence its catalytic properties. Notably, this trend in CO FEs was qualitatively reproduced by the corresponding, product-specific partial current densities (CDs) toward CO, since performing a CV treatment and replacing the electrolyte before the potential hold led to a current of ≈0.9 mA/cm^2^, whereas skipping the EE step resulted in an ≈2-fold lower current (see Figure 6). Moreover, as with the FEs, the CO CDs in this study are systematically lower than those reported by Chauhan et al. (e.g., for the CV treatment sample, ≈0.9 here vs ≈1.4 mA/cm^2^ in ref (21); see Figure S11b), possibly (again) due to the decrease in current density caused by the higher concentration of Cu on these samples’ surface.

**Figure 7:**
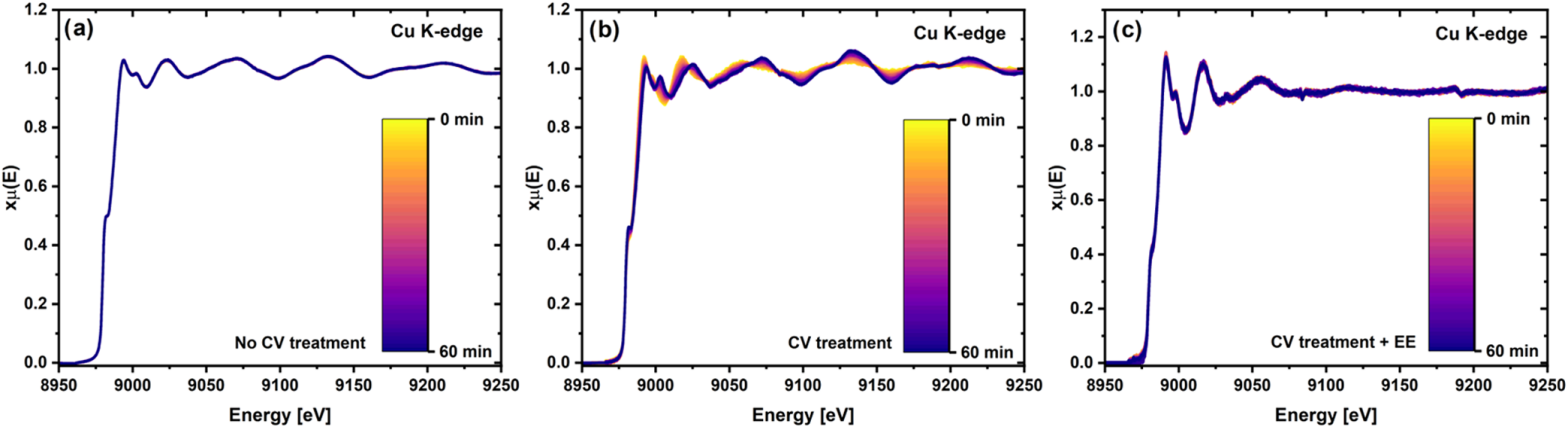
*Operando* GIXAS spectra at the Cu K-edge for a 100 μg_catalyst_/cm^2^ AuCu aerogel working electrode in a CO_2_-saturated 0.5 M KHCO_3_ at −0.5 V vs RHE for 60 min undergoing (a) no CV treatment, (b) CV treatment, or (c) CV treatment + EE prior to the potential hold.

In addition to these CO_2_RR tests in the laboratory, we also performed *operando* XAS measurements of the corresponding samples to infer more about the structural and/or electronic changes undergone during these potential holds. Focusing first on the Cu K-edge (see Figure 7), the “no CV treatment” sample is completely reduced by the time −0.5 V vs RHE is reached prior to the beginning of the potential hold and corresponds to the metallic AuCu alloy with the EXAFS fit yielding CNs of 6.2 ± 0.6 for Cu and 1.7 ± 0.3 for Au (see Figure S12 and Table S5). Note that the absence of Cu oxide contributions to these spectra stems from the fact that the XAS measurement was preceded by a potential scan to −0.5 V vs RHE at which the initial oxide gets reduced to Cu^0^.^47,48^ Moreover, this composition does not appear to change in the course of the 60 min long potential hold (Figure 7a). This lack of changes is also applicable for the “CV treatment + EE” sample (see Figure 7c), for which the EXAFS fit of the Cu K-edge spectrum reveals a stable AuCu alloy phase with a higher Au content resulting in a CN of 3.8 ± 0.8 for Cu and 6.6 ± 1.3 for Au (see Figure S12 and Table S5), whereby this enrichment in Au can explain the corresponding enhancement in CO selectivity and activity. Furthermore, IL-TEM measurements performed by Chauhan et al.^21^ on the AuCu aerogel before and after a CO_2_RR experiment, conducted under the same conditions as for the “CV treatment + EE” sample, revealed no significant structural changes, further supporting the stability of the catalyst under reaction conditions.

In contrast to this compositional stability, in the “CV treatment” experiment in which the electrolyte was not exchanged, drastic variations were observed at the Cu K-edge throughout the potential hold (Figure 7b). Following MCR analysis of the recorded data, two different components were identified (Figure S13). EXAFS fitting of the initially predominant component revealed that it consists of a AuCu alloy phase with a high Au content stemming from the CV treatment, with CNs of 3.8 ± 0.8 for Cu and 6.9 ± 1.4 for Au (Figure S12 and Table S5). As the experiment progressed, a second AuCu alloy phase with a higher Cu content emerged, in this case with CNs of 8.8 ± 0.8 for Cu and 2.6 ± 0.5 for Au (Figure S12 and Table S5). This result is consistent with the earlier hypothesis that the lower FE and partial CD toward CO compared to the “CV treatment + EE” experiment could be due to the redeposition of Cu. Notably, even if these two components are identified as AuCu phases, we cannot draw any unambiguous conclusions from this result if the deposited Cu is alloying with Au in the course of the potential hold.

As for the spectra recorded at the Au L_3_-edge for the three different potential holds, no changes were observed in any of the cases (see Figure S14). The EXAFS fits of the three samples’ spectra featured in Figure S15 identified the corresponding components as metallic AuCu alloys with a low, average CN toward Cu of ≈1.0 (see Table S6). This suggests that the Cu atoms deposited during the “CV treatment” experiment are unlikely to alloy with the Au atoms, at least not to an extent detectable by XAS. Instead, we hypothesize that these Cu atoms are mainly deposited as separate Cu side phases (*vide supra*).

To further verify the surface changes undergone by these samples during the potential holds (or absence thereof), at the end of each CO_2_RR measurement, we recorded a positive-going LSV at a scan rate of 20 mV/s from the holding potential of −0.5 to 1.7 V vs RHE, followed by CVs at the same scan rate between 0.1 and 1.7 V vs RHE (see Figure S16). Without CV treatment, a large oxidative current corresponding to the stripping of its abundant Cu oxide side phases was observed. As for the CV-treated samples, a significant, similar stripping current was observed for the experiment without electrolyte exchange, again corresponding to the oxidation of the Cu redeposited during the CO_2_RR test and thus confirming this Cu deposition suggested by the *operando* XAS results. Finally, when the electrolyte was exchanged, a stable CV with only a slight upward shift in the first cycle was observed—an additional oxidative current that is probably caused by the oxidation of CO_2_RR products adsorbed on the catalyst’s surface.^49^

In summary, the CV treatment significantly improved the CO selectivity and activity of the AuCu aerogel catalyst by enriching the surface with Au. This performance enhancement was especially successful when this CV treatment was accompanied by the exchange of the electrolyte prior to the CO_2_RR test, which prevented Cu redeposition during the potential hold. In contrast, the lack of electrolyte exchange resulted in Cu redeposition and a somewhat reduced CO_2_-to-CO selectivity and current density.

## Conclusions

By combining electrochemical measurements with *operando* GIXAS measurements, this study demonstrates that a CV treatment performed on a AuCu aerogel prior to its use as a CO_2_-reduction catalyst effectively modified its composition by removing Cu oxide side phases and enriching its surface with Au. This led to a significant improvement of the catalyst’s electrochemical performance, translating into an increase in the FE for CO from 23% for the unmodified material to 81% for the CV-treated catalyst with EE. This finding highlights the ability to adjust the AuCu surface composition in situ within the electrochemical cell before the potential hold, thereby boosting CO activity. Notably, this CV treatment offers a practical advantage as it eliminates the need for synthesizing AuCu catalysts with an inherently Au-rich surface. Furthermore, it enables the reactivation of catalysts that have degraded over time due to Cu oxidation, restoring their surface composition and CO selectivity.

Moreover, the removal of the Cu ions dissolved in the electrolyte during the CV treatment preceding the CO_2_-electroreduction test plays a critical role for this performance enhancement, since in the absence of electrolyte exchange, the progressive deposition of these Cu ions on the aerogel’s surface causes a significant decay of the CO selectivity and current density. Most importantly, these results perfectly portray the extended compositional insights that can be gained by combining electrochemical measurements with time-resolved (GI)XAS and open the door to further enhancing the CO_2_RR-performance of multimetallic catalysts by tuning their composition through CV treatments similar to the one applied herein. Future work will focus on evaluating this activation process for the CuAu aerogel in an electrolyzer cell and conducting long-term stability studies at high current densities (>100 mA/cm^2^).

## Supporting Information Available

Fourier-transformed fitted EXAFS spectra plus fitting data; STEM images and EDS elemental maps; CV of the AuCu working electrode with different potential boundaries; comparison CV to previous study;^21^ examples for how charges were integrated in Figure 2b; comparison of operando XAS spectra with spectra of references; comparison of activity and selectivity data to previous study;^21^ and difference of operando GIXAS spectra from components 1 and 2 during CV treatment at the Au L3-edge, operando GIXAS spectra, LSV, and CVs after potential holds (PDF)

## Abbreviations

CO: carbon monoxide
FE: faradaic efficiency
RHE: reversible hydrogen electrode
CV: cyclic voltammetry
CO_2_RR: carbon dioxide reduction reaction
ECSA: electrochemical active surface area
UPD: underpotential deposition
GI: grazing incidence
XAS: X-ray absorption spectroscopy
OCV: open circuit voltage
LSV: linear sweep voltammetry
EXAFS: extended X-ray absorption fine structure
HAADF STEM: high-angle annular dark-field scanning transmission electron microscopy
EDS: energy-dispersive X-ray spectroscopy
CN: coordination number
EE: electrolyte exchange
CD: current density
