# Supplementary material for: Monitoring the Activation of a AuCu Aerogel CO2-Reduction Electrocatalyst via Operando XAS
Source: Langmuir. 2025 Apr 25;41(17):11026–36. doi: 10.1021/acs.langmuir.5c00662 (PMC12060650; doi:10.1021/acs.langmuir.5c00662)
Supplement: Supplementary file 1 — la5c00662_si_001.pdf [file la5c00662_si_001.pdf]

Supporting Information

Monitoring the Activation of a AuCu Aerogel CO_2-Reduction Electrocatalyst via *Operando* XAS

Maximilian Winzely[1], Adam H. Clark[2], Deema Balalta[3], Piyush Chauhan[1], Paul M. Leidinger[1], Meriem Fikry[1], Tym de Wild[1], Maximilian Georgi[4], Alexander Eychmüller[4], Sara Bals[3], Thomas J. Schmidt[1,5], Juan Herranz[1*]

[1] PSI, Center for Energy and Environmental Science, CH-5232 Villigen, Switzerland

[2] PSI, Center for Photon Science, CH-5232 Villigen PSI, Switzerland

[3] University of Antwerp, Electron Microscopy for Materials Science, BE-2020 Antwerpen, Belgium

[4] Technische Universität Dresden, Physical Chemistry, DE-01062 Dresden, Germany

[5] ETH Zürich, Institute for Molecular Physical Science, CH-8093 Zürich, Switzerland

Table of Content:

Figure S1	**S2**	Figure S9	**S8**
STable S1	**S2**	Table S4	**S9**
Figure S2	**S3**	Figure S10	**S9**
Table S2	**S3**	Figure S11	**S10**
Figure S3	**S4**	Figure S12	**S11**
Figure S4	**S4**	Table S5	**S12**
Figure S5	**S5**	Figure S13	**S12**
Figure S6	**S5**	Figure S14	**S12**
Figure S7	**S6**	Figure S15	**S13**
Table S3	**S7**	Table S6	**S14**
Figure S8	**S8**	Figure S16	**S14**

Content of the Supporting Information:

Number of pages: 14
Number of figures: 16
Number of tables: 6

*Corresponding author: juan.herranz@psi.ch

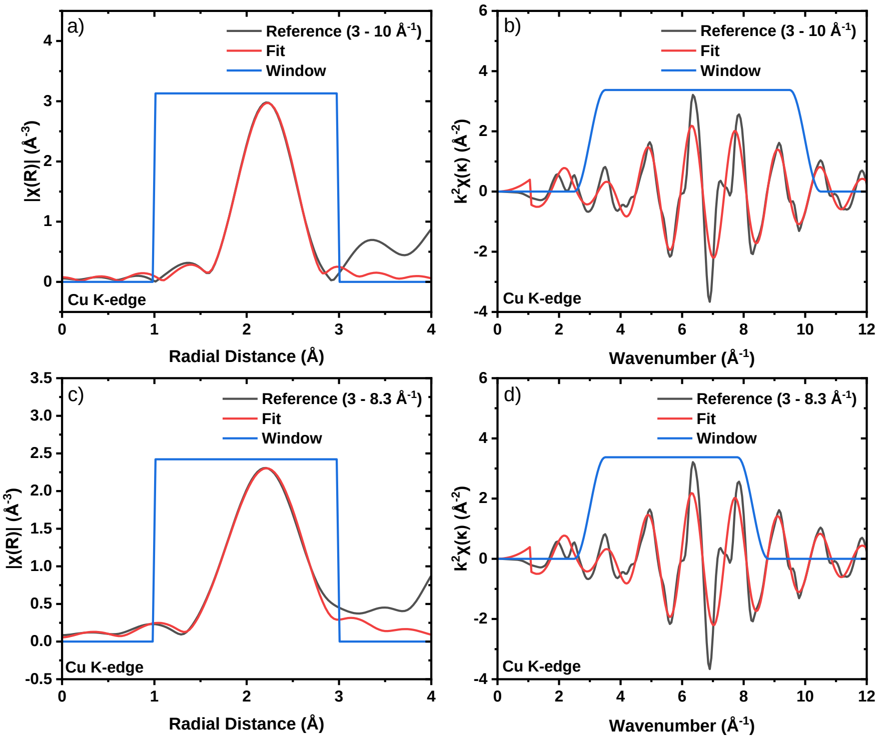

Figure S1. k^2-space and R-space EXAFS fits at the Cu K-edge of the Cu reference foil that was used for energy calibration with k-window ranges of 3 – 10 and 3 – 8.3 $Å^{-1}$ (panels 'a' and 'b' vs. 'c' and 'd', respectively). The data (black line), fit (red line) as well as the Hanning fitting window (blue line) are shown for the R-space in the left panel and for the k-space in the right panel.

Table S1. Fitting parameters of the EXAFS fits in Figure S1 at the Cu K-edge of the Cu reference foil that was used for energy calibration with 3 – 10 and 3 – 8.3 $Å^{-1}$ as the k-window.

**Comp.**	**$CN_{Cu1.1}$ [-]**	**S_0^2 [-]**	**σ^2_{Cu} [10^{-3} $Å^{-2}$]**	**ΔE_0 [eV]**	**R_{Cu} [Å]**
Reference (3 – 10 $Å^{-1}$)	12	0.872 ± 0.063	8.9 ± 0.6	4.5 ± 0.7	2.560 ± 0.005
Reference (3 – 8.3 $Å^{-1}$)	12	0.858 ± 0.109	8.7 ± 0.1	4.5 ± 1.1	2.560 ± 0.008

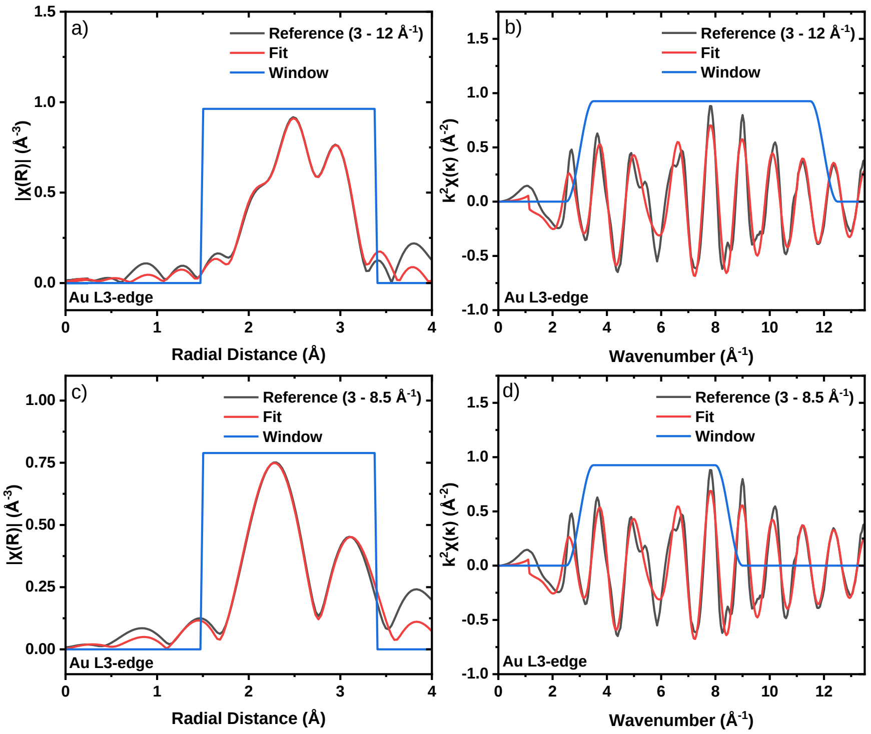

Figure S2. k^2-space and R-space EXAFS fits at the Au L_3-edge of the Au reference foil that was used for energy calibration with k-window ranges of 3 – 12 and 3 – 8.5 $Å^{-1}$ (for panels 'a' and 'b' vs. 'c' and 'd', respectively). The data (black line), fit (red line) as well as the Hanning fitting window (blue line) are shown for the R-space in the left panel and for the k-space in the right panel.

Table S2. Fitting parameters of the EXAFS fits in Figure S2 at the Au L_3-edge of the Au reference foil that was used for energy calibration with 3 – 12 and 3 – 8.5 $Å^{-1}$ as the k-window.

**Comp.**	**$CN_{Au1.1}$ [-]**	**S_0^2 [-]**	**σ^2_{Au} [10^{-3} $Å^{-2}$]**	**ΔE_0 [eV]**	**R_{Au} [Å]**
Reference (3 – 12 $Å^{-1}$)	12	0.794 ± 0.028	7.9 ± 0.3	4.9 ± 0.3	2.884 ± 0.002
Reference (3 – 8.5 $Å^{-1}$)	12	0.814 ± 0.056	8.3 ± 0.8	4.8 ± 0.5	2.884 ± 0.004

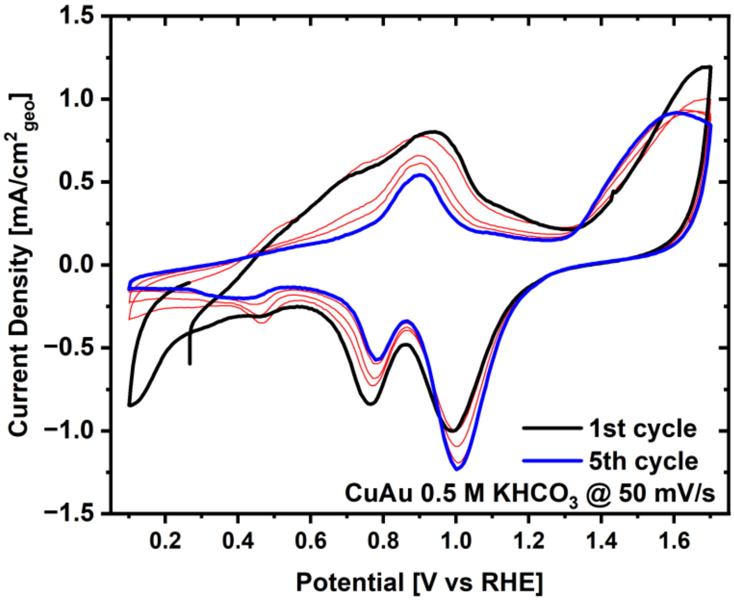

Figure S3. CVs recorded in the study by Chauhan et al. [1] during the CV treatment of a 100 $\mu g_{catalyst}/cm^2$ AuCu aerogel working electrode at a scan rate of 50 mV/s in CO_2-satutrated 0.5 M $KHCO_3$ between 0.1 and 1.7 V vs RHE. The first cycle of the CV treatment is illustrated as a black line while the last cycle is depicted as a blue line.

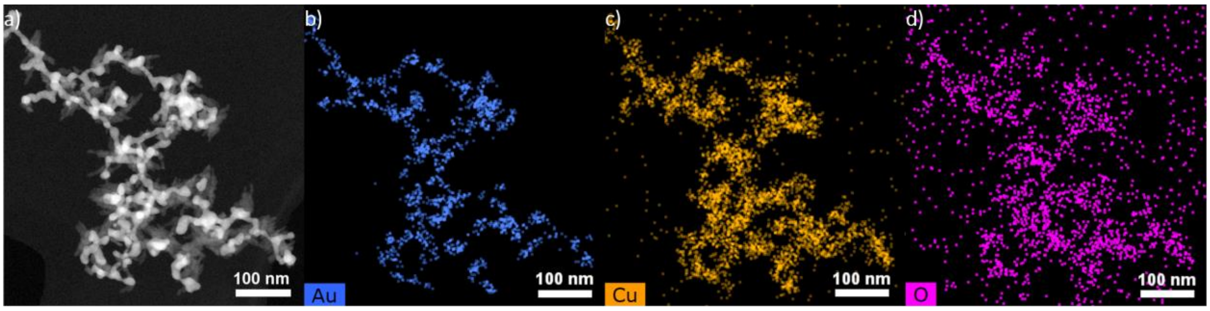

Figure S4. a) HAADF STEM image of the synthesized CuAu aerogel and its EDS elemental maps of b) gold, c) copper, d) oxygen.

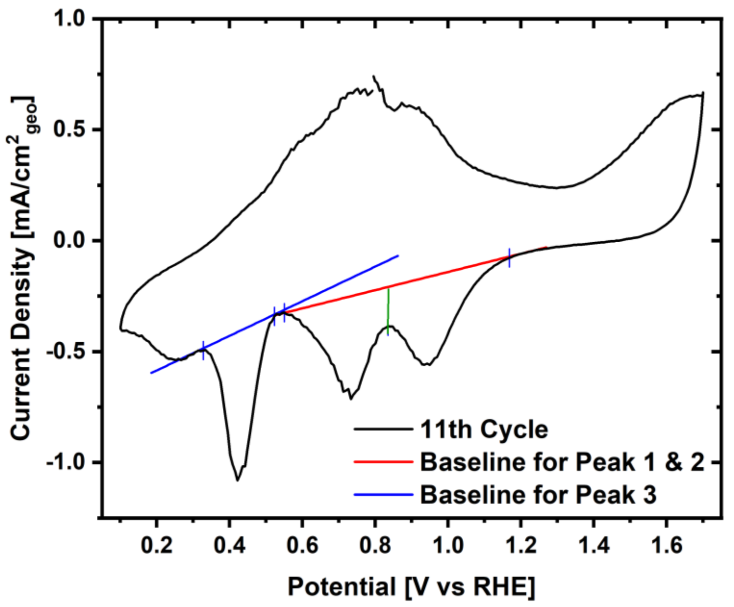

Figure S3. 11th CV recorded during the CV treatment shown in Figure 2, including the baselines for the integration of the charge of peaks 1 and 2 (red line) and for peak 3 (blue line). The integration boundary between peak 1 and 2 is depicted as the green line.

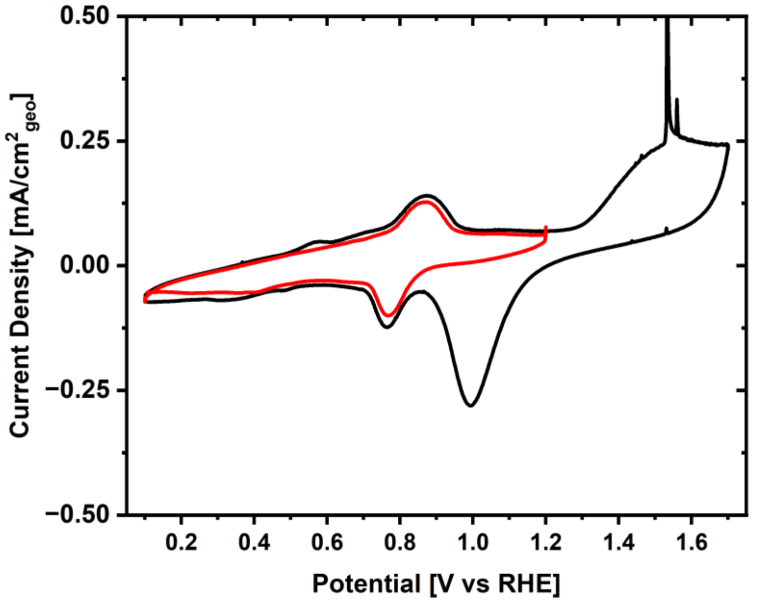

Figure S4. CVs recorded at a scan rate of 20 mV/s on a AuCu working electrode with a loading of 100 $\mu g_{catalyst}/cm^2$ in CO_2-saturated 0.5 M $KHCO_3$ electrolyte, where a potential range of 0.1 to 1.2 V vs. RHE was used for the CV depicted with the red line and a potential range of 0.1 to 1.7 V vs RHE was used for the CV illustrated with the black line.

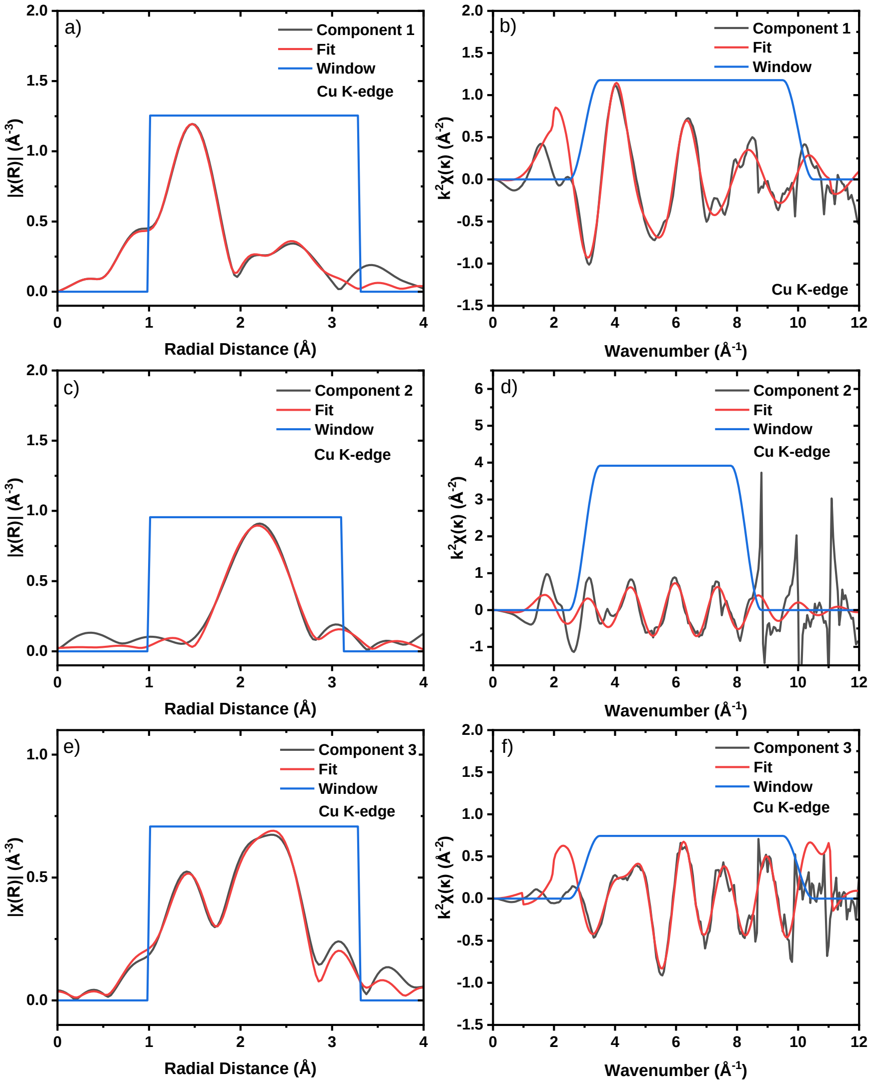

Figure S7. *Operando* EXAFS k^2-space and R-space fits at the Cu K-edge of the three components that were used to describe the set of recorded XA-spectra in Figure 3 b) on a AuCu working electrode with a loading of 100 $\mu g_{catalyst}/cm^2$ in CO_2-saturated 0.5 M $KHCO_3$ electrolyte (for panels 'a', 'b' and 'c' vs. 'd', 'e' and 'f', respectively). The data (black line), fit (red line) as well as the Hanning fitting window (blue line) are shown for the R-space in the left panel and for the k-space in the right panel.

Table S3. Fitting parameters of the *operando* EXAFS fit (Figure S5) at the Cu K-edge of the three components that were used to describe the set of recorded XA-spectra in Figure 3 b) on a AuCu working electrode with a loading of 100 $\mu g_{catalyst}/cm^2$ in CO_2-saturated 0.5 M $KHCO_3$ electrolyte.

**Comp.**	**$CN_{Cu1.1}$ [-]**	**$CN_{O1.1}$ [-]**	**$CN_{O1.3}$ [-]**	**$CN_{CuCu1.1}$ [-]**	**$CN_{CuCu1.2}$ [-]**	**$CN_{Au1.1}$ [-]**	**$\sigma^2_{Cu/Au}$ [10^{-3}Å^{-2}]**	**σ^2_{O} [10^{-3}Å^{-2}]**	**ΔE_0 [eV]**	**$R_{Cu1.1}$ [Å]**	**$R_{O1.1}$ [Å]**	**$R_{O1.3}$ [Å]**	**$R_{Cucu1.1}$ [Å]**	**$R_{Cucu1.2}$ [Å]**	**R_{Au} [Å]**
1	-	3.2 ± 0.6	1.6 ± 0.3	1.7 ± 0.3	1.7 ± 0.3	0.9 ± 0.2	9.3 ± 1.9	3.5 ± 0.7	0.1 ± 7.3	-	1.96 ± 0.04	2.78 ± 0.04	2.90 ± 0.10	3.08 ± 0.10	2.70 ± 0.12
2	6.9 ± 1.4	-	-	-	-	2.7 ± 0.5	14.3 ± 2.9	-	-0.9 ± 6.2	2.56 ± 0.05	-		-		2.70 ± 0.21
3	1.9 ± 0.4	1.4 ± 0.3	0.7 ± 0.1	1.4 ± 0.3	1.4 ± 0.3	-	4.2 ± 0.8	6.0 ± 1.2	3.7 ± 5.6	2.56 ± 0.03	1.96 ± 0.05	2.78 ± 0.05	2.90 ± 0.12	3.08 ± 0.12	-

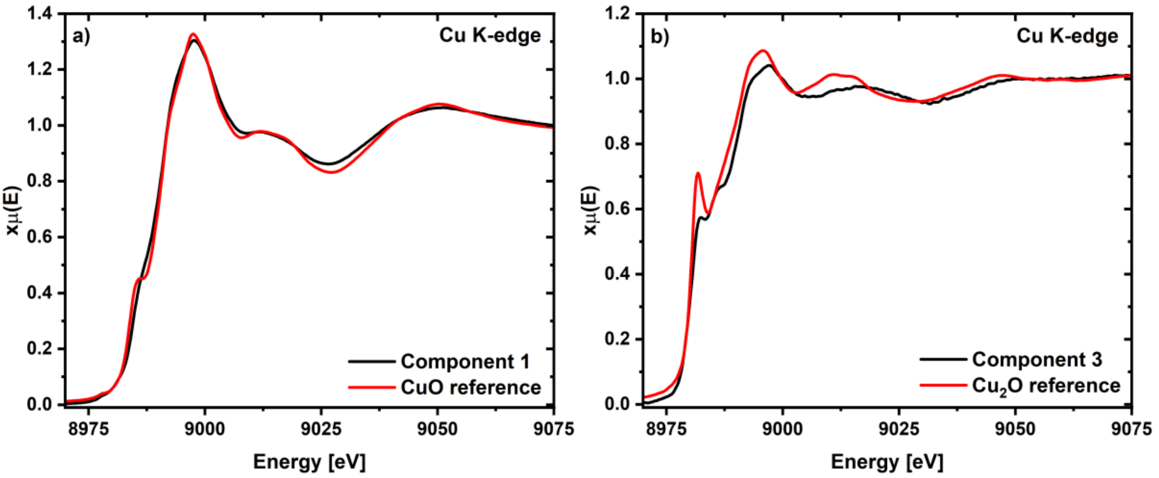

Figure S8. Comparison of the *operando* XAS spectrum at the Cu K-edge of a) component 1 with a CuO reference spectrum and b) component 3 with a Cu_2O reference spectrum, which were found to be Cu phases of the AuCu aerogel during the CV treatment by using SVD, SIMPLISMA and MCR analysis.

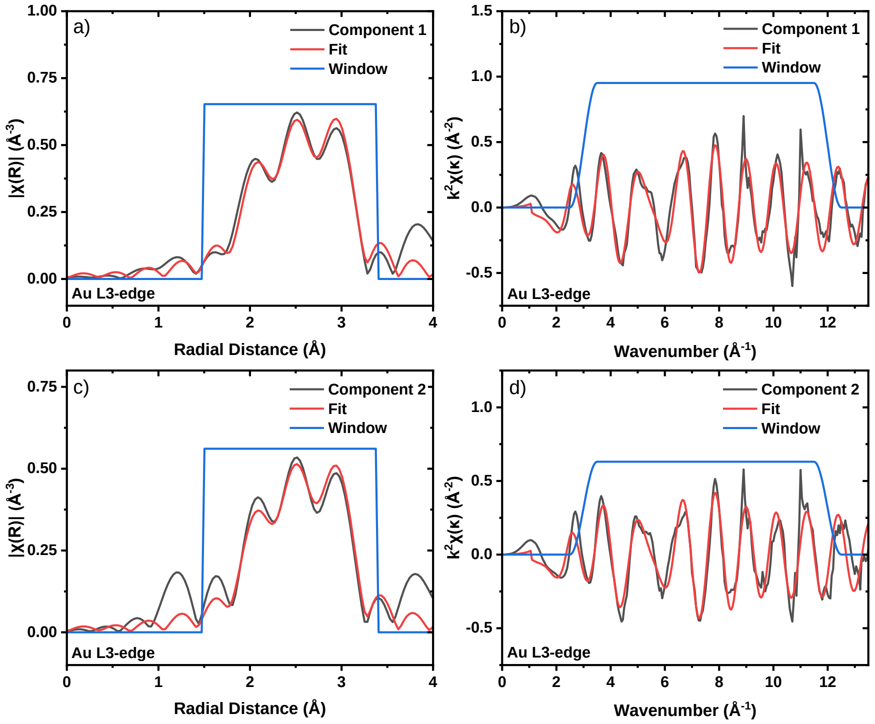

Figure S9. *Operando* EXAFS k^2-space and R-space fits at the Au L_3-edge of the two components that were used to describe the set of recorded XA-spectra in Figure 3 d) on a AuCu working electrode with a loading of 100 $\mu g_{catalyst}/cm^2$ in CO_2-saturated 0.5 M $KHCO_3$ electrolyte. The data (black line), fit (red line) as well as the Hanning fitting window (blue line) are shown for the R-space in the left panel and for the k-space in the right panel.

Table S4. Fitting parameters of the *operando* EXAFS fit (Figure S7) at the Au L3-edge of the two components that were used to describe the set of recorded XA-spectra in Figure 3 d) on a AuCu working electrode with a loading of 100 $\mu g_{catalyst}/cm^2$ in CO_2-saturated 0.5 M $KHCO_3$ electrolyte.

Comp.	$CN_{Au1.1}$ [-]	$CN_{Cu1.1}$ [-]	$\sigma^2_{Au/Cu}$ [10^{-3}Å^{-2}]	ΔE_0 [eV]	R_{Au} [Å]	R_{Cu} [Å]
1	8.8 ± 1.0	0.7 ± 0.1	7.6 ± 0.1	4.5 ± 0.8	2.884 ± 0.005	2.70 ± 0.02
2	7.3 ± 1.0	0.5 ± 0.1	7.4 ± 0.9	4.3 ± 1.0	2.884 ± 0.006	2.70 ± 0.03

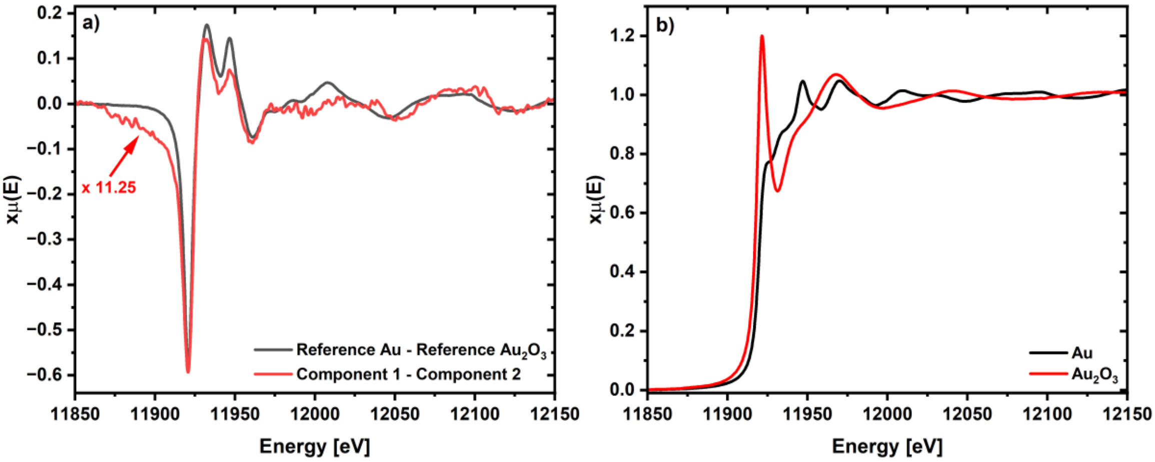

Figure S10.a) Difference of the two *operando* XA spectra at the Au L_3-edge shown in Figure 3e compared to the difference of the XA-spectra of a reference Au foil and a reference Au_2O_3 powder which are depicted in panel b).

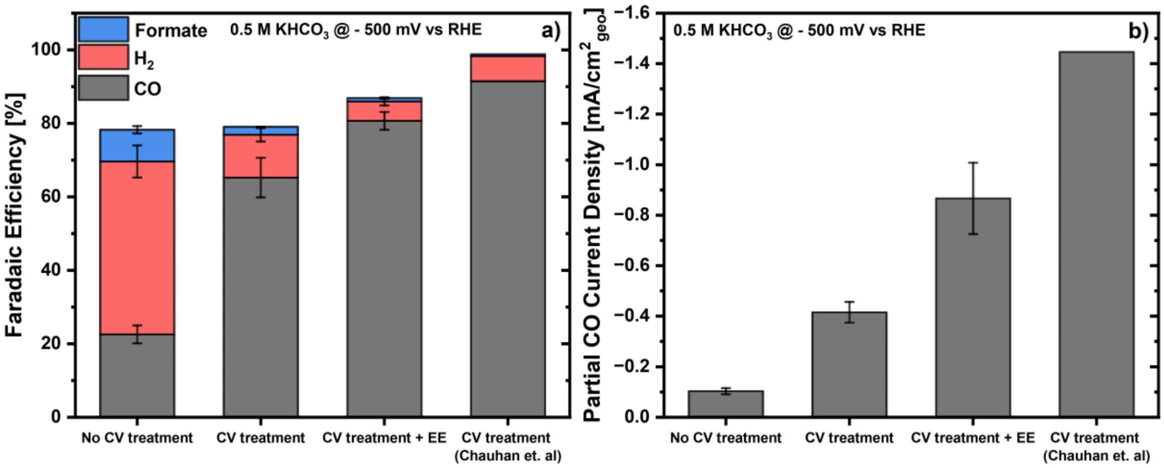

Figure S11. a) FEs for CO, H_2 and formate production and b) partial CDs for CO during the electrochemical reduction of CO_2 in CO_2-saturated 0.5 M $KHCO_3$ for a 100 $\mu g_{catalyst}/cm^2$ AuCu aerogel working electrode undergoing No CV treatment, CV treatment or CV treatment + EE prior to the potential hold at – 0.5 V vs RHE. Additionally, as comparison the result from our previous study is illustrated for a AuCu working electrode with the same loading using the same electrolyte but undergoing only 5 CVs at 50 mV/s between 0.1 and 1.7 V vs RHE as CV treatment before the potential hold. [1]

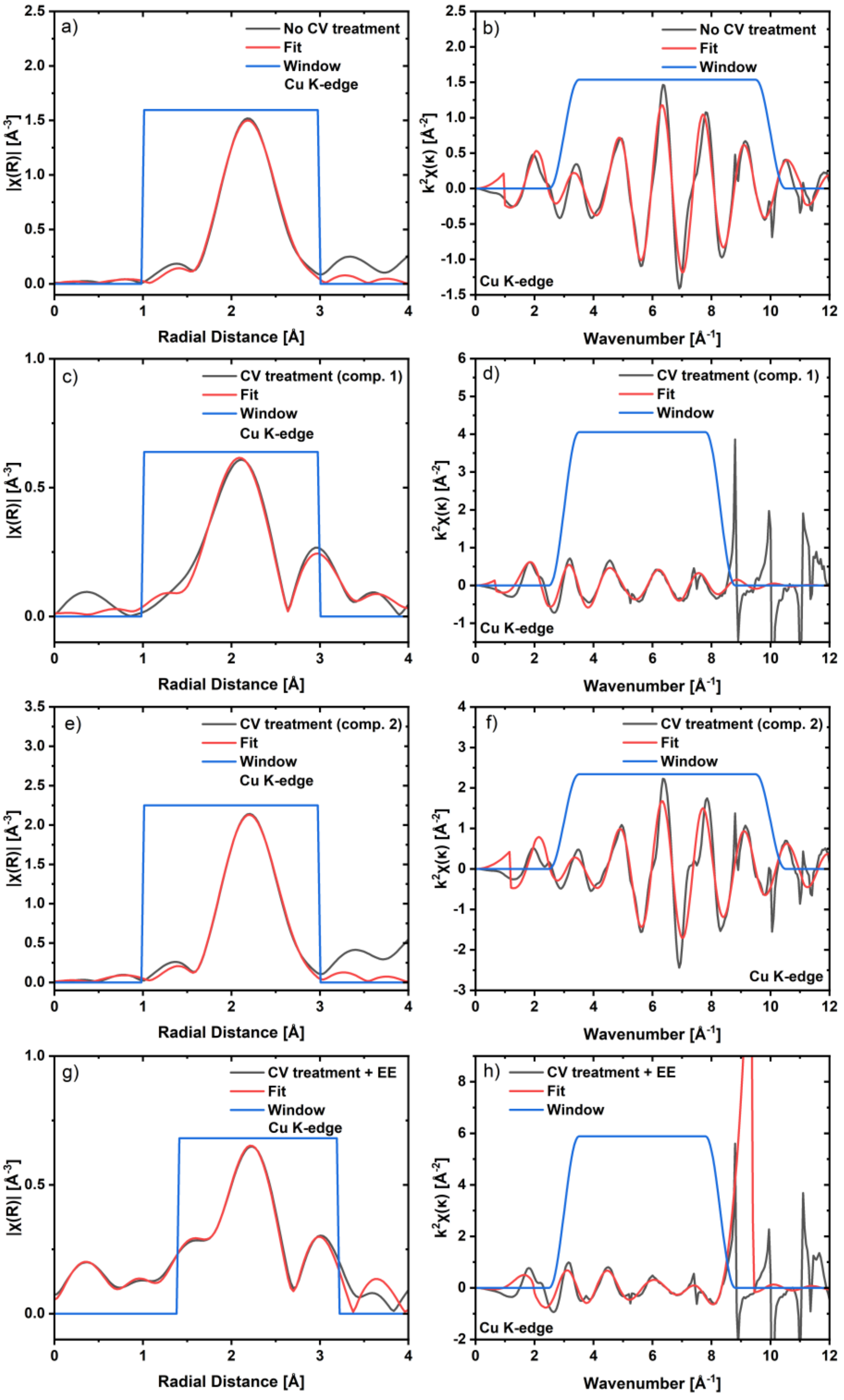

Figure S12. *Operando* EXAFS k^2-space and R-space fits at the Cu K-edge during the potential hold at -0.5 V vs RHE using a AuCu working electrode with a loading of 100 $\mu g_{catalyst}/cm^2$ in CO_2-saturated 0.5 M $KHCO_3$ electrolyte which was undergoing no CV treatment (a and b), CV treatment were two components could be identified during the potential hold (c, d, e and f) and CV treatment + EE (g and h) prior to the potential hold. The data (black line), fit (red line) as well as the Hanning fitting window (blue line) are shown for the R-space in the left panel and for the k-space in the right panel.

Table S5. Fitting parameters of the *operando* EXAFS fit (Figure S9) at the Cu K-edge during the potential hold at – 0.5 V vs RHE using a AuCu working electrode with a loading of 100 $\mu g_{catalyst}/cm^2$ in CO_2-saturated 0.5 M $KHCO_3$ electrolyte.

Comp.	$CN_{Cu1.1}$ [-]	$CN_{Au1.1}$ [-]	$\sigma^2_{Cu/Au}$ [10^{-3}Å^{-2}]	ΔE_0 [eV]	R_{Cu} [Å]	R_{Au} [Å]
No CV treatment	6.2 ± 0.6	1.7 ± 0.3	9.2 ± 1.0	3.7 ± 1.2	2.560± 0.007	2.70 ± 0.03
CV treatment (component 1)	3.8 ± 0.8	6.9 ± 1.4	19.9 ± 4.0	1.8 ± 5.9	2.56 ± 0.04	2.70 ± 0.12
CV treatment (component 2)	8.8 ± 0.8	2.6 ± 0.5	8.5 ± 0.9	5.5 ± 1.2	2.560 ± 0.006	2.70 ± 0.03
CV treatment + EE	3.8 ± 0.8	6.6 ± 1.3	15.0 ± 3.0	-1.9 ± 10.0	2.56 ± 0.13	2.70 ± 0.17

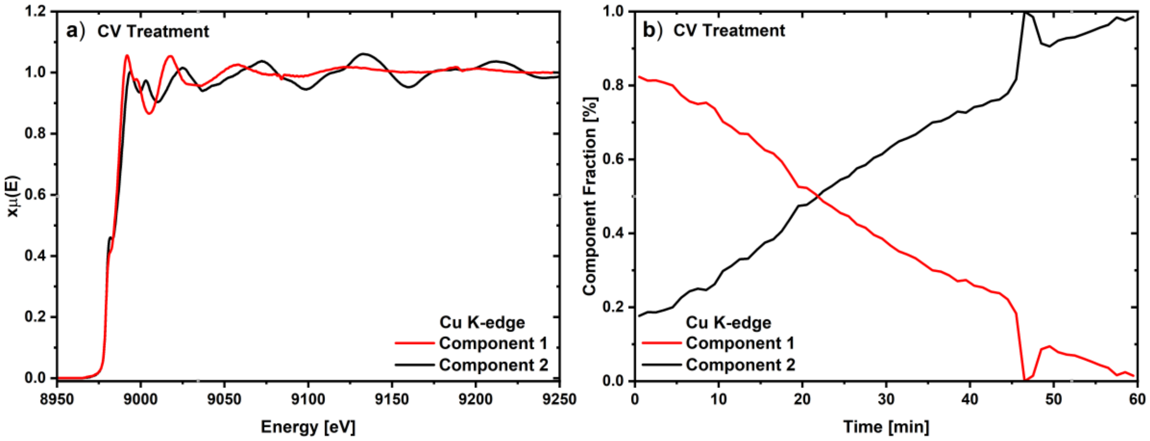

Figure S13. a) *Operando* GIXA spectra of two components that were identified to describe the dataset of XA spectra which were recorded during the potential hold at – 0.5 V vs RHE using a AuCu working electrode with a loading of 100 $\mu g_{catalyst}/cm^2$ in CO_2-saturated 0.5 M $KHCO_3$ electrolyte and the corresponding b) MCR analysis.

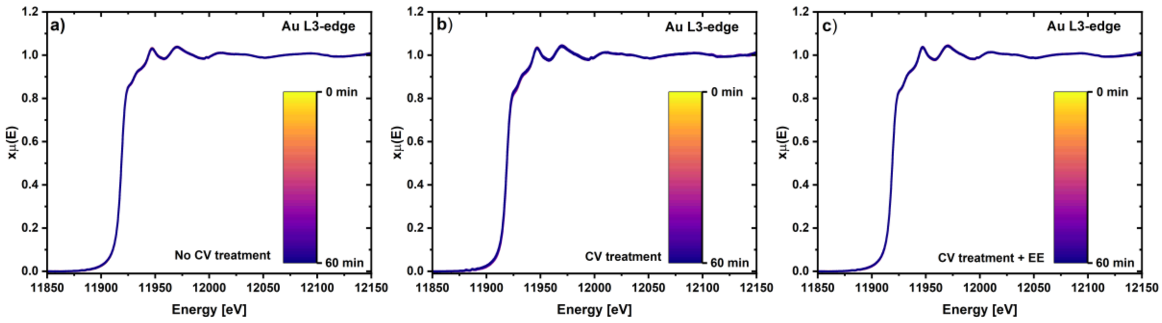

Figure S14. *Operando* GIXAS spectra at the Au L3-edge for a 100 $\mu g_{catalyst}/cm^2$ AuCu aerogel working electrode in CO_2-saturated 0.5 M $KHCO_3$ at – 0.5 V vs RHE for 60 minutes undergoing a) No CV treatment, b) CV treatment or c) CV treatment + EE prior to the potential hold.

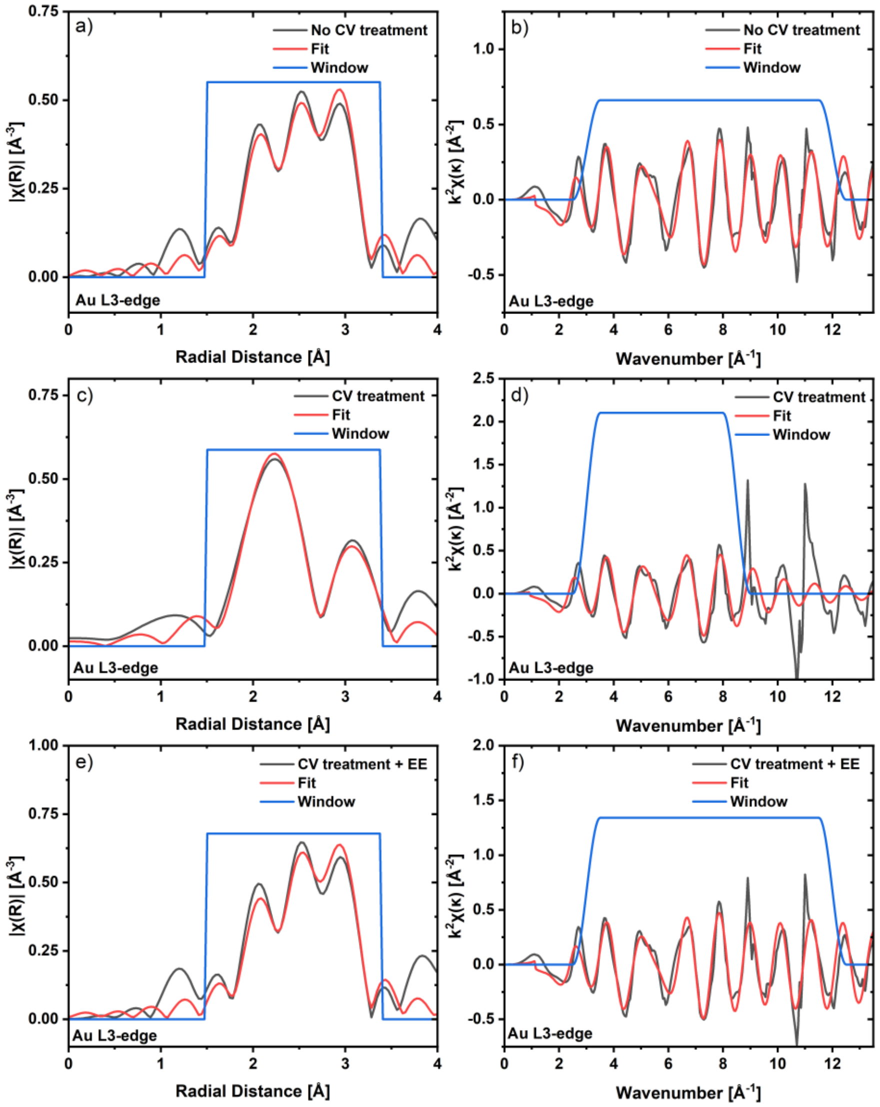

Figure S15. *Operando* EXAFS k^2-space and R-space fits at the Au L3-edge during the potential hold at – 0.5 V vs RHE using a AuCu working electrode with a loading of 100 $\mu g_{catalyst}/cm^2$ in CO_2-saturated 0.5 M $KHCO_3$ electrolyte which was undergoing no CV treatment (a and b), CV treatment (c and d) and CV treatment + EE (e and f) prior to the potential hold. The data (black line), fit (red line) as well as the Hanning fitting window (blue line) are shown for the R-space in the left panel and for the k-space in the right panel.

Table S6. Fitting parameters of the *operando* EXAFS fit (Figure S12) at the Au L3-edge during the potential hold at – 0.5 V vs RHE using a AuCu working electrode with a loading of 100 $\mu g_{catalyst}/cm^2$ in CO_2-saturated 0.5 M $KHCO_3$ electrolyte.

Comp.	$CN_{Au1.1}$ [-]	$CN_{Cu1.1}$ [-]	$\sigma^2_{Au/Cu}$ [10^{-3} Å^{-2}]	ΔE_0 [eV]	R_{Au} [Å]	R_{Cu} [Å]
No CV treatment	7.6 ± 0.8	0.9 ± 0.2	7.4 ± 0.7	4.7 ± 0.8	2.884 ± 0.004	2.70 ± 0.01
CV treatment	9.8 ± 2.0	1.0 ± 0.2	11.6 ± 2.3	3.3 ± 2.5	2.88 ± 0.04	2.70 ± 0.11
CV treatment + EE	8.2 ± 1.1	0.8 ± 0.2	6.8 ± 0.9	4.8 ± 1.0	2.884 ± 0.006	2.70 ± 0.02

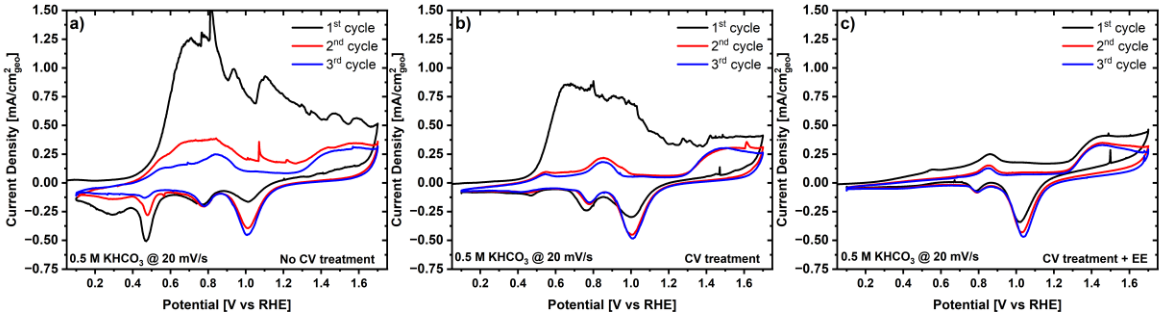

Figure S16. LSV and CVs at a scan rate of 20 mV/s after the potential hold at – 0.5 V vs RHE using a AuCu working electrode with a loading of 100 $\mu g_{catalyst}/cm^2$ in CO_2-saturated 0.5 M $KHCO_3$ electrolyte which was undergoing no CV treatment (a), CV treatment (b) and CV treatment + EE (c) prior to the potential hold.

References

1. Chauhan, P., et al., *Impact of Surface Composition Changes on the CO2-Reduction Performance of Au–Cu Aerogels.* Langmuir, 2024.
